# Carbonyl-Terminated Quinoidal Oligothiophenes as p-Type Organic Semiconductors

**DOI:** 10.3390/ma13133020

**Published:** 2020-07-06

**Authors:** Takato Asoh, Kohsuke Kawabata, Kazuo Takimiya

**Affiliations:** 1 Department of Chemistry, Graduate School of Science, Tohoku University, Aoba-ku, Sendai, Miyagi 980–8578, Japan; takao.aso.r2@dc.tohoku.ac.jp (T.A.); kohsuke.kawabata.b2@tohoku.ac.jp (K.K.); 2 RIKEN Center for Emergent Matter Science, Wako, Saitama 351–0198, Japan

**Keywords:** organic field-effect transistor, p-type organic semiconductor, quinoidal oligothiophene, molecular design, organic synthesis, theoretical calculation

## Abstract

A series of quinoidal oligothiophenes terminated with carbonyl groups (*n*TDs, *n* = 2–4) are studied as p-type organic semiconductors for the active materials in organic field-effect transistors (OFETs) both by the theoretical and experimental approaches. The theoretical calculations clearly show their high-lying highest occupied molecular orbital (HOMO) energy levels (*E*_HOMO_s), small reorganization energies for hole transport (*λ*_hole_s), and large contribution of sulfur atoms to HOMOs, all of which are desirable for p-type organic semiconductors. Thus, we synthesized *n*TDs from the corresponding aromatic oligothiophene precursors and then evaluated their physicochemical properties and structural properties. These experimental evaluations of *n*TDs nicely proved the theoretical predictions, and the largest 4TDs in the series (4,4′′′-dihexyl- and 3′,4,4″,4′′′-tetrahexyl-5*H*,5′′′*H*-[2,2′:5′,2″:5″,2′′′-quaterthiophene]-5,5′′′-dione) can afford solution-processed OFETs showing unipolar p-type behaviors and hole mobility as high as 0.026 cm^2^ V^−1^ s^−1^.

## 1. Introduction

The last several decades have witnessed a rapid progress of organic electronic devices including organic field-effect transistors (OFET) [1], organic photovoltaics [2], and organic light-emitting diodes (OLED) [3], in which π-conjugated organic molecules are utilized as the active materials. Such organic molecules, often referred as organic semiconductors, play key roles in the electronic devices to determine the device performances, and thus the development of superior organic semiconductors has been highly awaited. Among many organic semiconductors reported so far, oligothiophenes are one of the most classical yet important class of organic semiconductors [4,5]. The characteristic features of oligothiophenes are (1) rigid and coplanar backbone structures with small dihedral angles between the five-membered heteroaromatic rings; (2) effective π-conjugation through the coplanar backbone structures, resulting in a small energy gap between the highest occupied molecular orbital (HOMO) and the lowest unoccupied molecular orbital (LUMO); (3) tunable physicochemical properties by the chemical modifications, e.g., alteration of the conjugation length as well as introduction of substituents, and (4) the high polarizability of the sulfur atoms incorporated in the thiophene rings, which can contribute to enhancing the intermolecular electronic couplings in the solid state. These features have attracted many research groups to employ oligothiophenes as versatile building blocks in the development of new organic semiconductors [6].

The quinoidization of aromatic molecules is a powerful way to modify the electronic structure while keeping the molecular framework (σ-bond framework) of the given aromatic molecules; quinoidal molecules lose aromaticity and have a different bond-alternation path from that of the aromatic (benzenoid) counterpart, which generally reduces the HOMO–LUMO energy gap (*E_g_*). Furthermore, quinoidal molecules should have additional end groups that terminate the quinoidal conjugation path, and strong electron-withdrawing groups such as dicyanomethylene group have been frequently employed as the terminal groups. 7,7,8,8-Tetracyanoquinodimethane (TCNQ), long known as a strong electron acceptor, is a representative quinoidal molecule [7]. Quinoidal oligothiophenes [8,9] have also been studied since the 1980s initially as an electron acceptor [10] and then as n-type and ambipolar semiconductors [11,12,13,14,15]. Very recently, unipolar p-type OFET behaviors have been reported for quinoidal bithiophenes modified with fused-thiophene rings [16], which implies that an appropriate molecular design can realize p-type quinoidal oligothiophenes.

**Figure 1:**
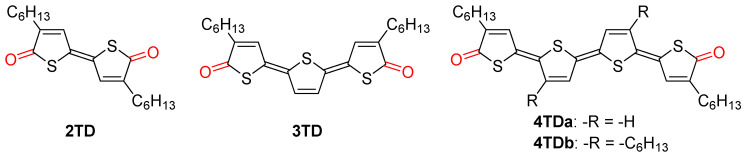
Molecular structures of carbonyl-terminated quinoidal oligothiophenes in this study.

In this study, we first discuss the potential of quinoidal oligothiophenes terminated with carbonyl groups with relatively weak electron-withdrawing character as p-type organic semiconductors, and then the synthesis and characterization of a series of quinoidal oligothiophenes, i.e., 4,4′-dihexyl-5*H*,5′*H*-[2,2′-bithiophenylidene]-5,5′-dione (2TD), 4,4″-dihexyl-5*H*,5″*H*-[2,2′:5′,2″-terthiophene]-5,5″-dione (3TD), 4,4′′′-dihexyl- (4TDa), and 3′,4,4″,4′′′-tetrahexyl-5*H*,5′′′*H*-[2,2′:5′,2″:5″,2′′′-quaterthiophene]-5,5′′′-dione (4TDb) (Figure 1), are reported. Then, we experimentally elucidate the electronic structure of 2TD and 4TDa in the solid state to further examine their potential as p-type organic semiconductors. Finally, solution-processed OFETs with 4TDs as the active materials are demonstrated to experimentally confirm the carrier transport properties of the present quinoidal oligothiophenes.

## 2. Materials and Methods

### 2.1. General Experimental Methods

All chemicals and solvents are of reagent grade unless otherwise indicated. Tetrahedrofuran (THF) was purified with a Glass Contour solvent purification system prior to use. FHF32EX-N-H white-light LEDs (Panasonic, Osaka, Japan) were used as room lights. NMR spectra were recorded on Avance III 500 (Bruker, Billerica, MA, USA) and JNM-ECA700 NMR spectrometers (JEOL, Tokyo, Japan). ^1^H and ^13^C NMR chemical shifts are reported in ppm relative to the signal for tetramethylsilane (0.00 ppm) or the signals for residual chloroform (7.27 ppm for ^1^H and 77.0 ppm for ^13^C NMR) as internal standards. High-resolution mass spectrometry (HRMS) was carried out with a JMS-T100GCV mass spectrometer (JEOL, Tokyo, Japan). Elemental analyses were carried out with a J-Science Lab JM10 CHN corder or a Yanaco MT-6 CHN corder, and values are reported as percentages. Melting points of 2TD, 3TD, 4TDa and 4TDb were determined as the peak temperature of differential scanning calorimetry (DSC) curves measured with a Shimadzu DSC-60 Plus at a heating and cooling rate of 10 °C min^−1^. UV-vis absorption spectra were measured with a UV-3600 plus spectrometer (Shimadzu, Kyoto, Japan). For the absorption spectra measurement, the sample solutions were prepared in a concentration of 0.01 mM in chloroform, and the thin-film samples were prepared by spin-coating a hot sample solution in chloroform (6 g/L) on a quartz glass at the spin-rate of 1000 rpm for 15 sec. Cyclic voltammetry was carried out with an Electrochemical Analyzer Model 612D (ALS, Tokyo, Japan) with the three electrode system consisting of a platinum disc working electrode (φ = 3 mm), an Ag/AgCl reference electrode, and a platinum wire counter electrode in a dichloromethane solution containing 0.05 mM of substrate and 0.1 M of tetrabutylammonium hexafluorophosphate at a scan rate of 10 mV s^−1^. All the potentials were calibrated with the half-wave potential of the ferrocene/ferrocenium redox couple measured under identical conditions [17]. Photoemission yield spectra were taken with an AC-2 photoemission yield spectrometer (Riken Keiki, Tokyo, Japan) in air by using thin films on indium-tin-oxide coated glass substrates which were prepared in the same condition used for the OFET fabrication. Single crystals of (*E*)-2TD and (*ZEZ*)-4TDa were prepared by the slow vapor diffusion of methanol into a chloroform solution and by slow solvent evaporation from a chloroform solution, respectively. Single-crystal X-ray analyses of 2TD and 4TDa were carried out on an R-AXIS RAPID (CuKα radiation, wavelength: 0.15418 nm, multilayer confocal optics, (Rigaku, Tokyo, Japan). The structures were solved by the SHELXT program [18]. Non-hydrogen atoms were refined anisotropically, and hydrogen atoms were included in the calculations but not refined by using the SHELXL program [19]. All calculations were carried out by using the crystallographic software package Olex2 (ver. 1.3.0, OlexSys, Durham, UK) [20]. X-ray diffractions of thin films of 4TDs spin-coated on trimethoxyoctylsilane-treated Si/SiO_2_ substrates were recorded on the Rigaku Ultima with a CuKα radiation (wavelength: 0.15418 nm). The Density functional theory (DFT) calculations were performed at the UB3LYP/6-311G** level by using a Gaussian 16 program package (ver. Rev C.01, Gaussian, Wallingford, CT, USA) [21]. Intermolecular electronic couplings (transfer integral, *t*) in different molecular dimers that were extracted from the single-crystal structures were calculated at the GGA PW91/TZP level by using the Amsterdam Density Functional (ADF) program [22]. Reproducibility of the experimental results in this study were confirmed by our hands; however, they might be influenced by different ambient conditions such as light source and temperature, which could alter the isomer ratio in the sample.

### 2.2. OFET Device Fabrication and Evaluation

Bottom-gate-top-contact OFET devices were fabricated on a heavily doped n^+^-Si (100) wafer with 200-nm thickness thermally grown SiO_2_ (*C*_i_ = 17.3 nF cm^−2^). The Si/SiO_2_ substrates were cleaned by ultrasonication with water for 3 min thrice and with acetone for 10 min and rinsed in boiling isopropanol, and then they were treated an UV-ozone cleaner for 30 min. The cleaned substrates and several drops of trimethoxyoctylsilane were placed together in a closed container and the container was kept in an oven at 80 °C overnight; then, the substrates were rinsed with boiling isopropanol. The *n*TDs were spin-coated from hot chloroform solutions (6 gL^−1^ for 4TDa and 36 gL^−1^ for 4TDb) at a spin rate of 6000 rpm for 10 sec in air. On the top of the thin films, 40-nm-thick gold drain and source contact electrodes with the channel width (*W*) and length (*L*) of 1500 and 100 μm, respectively, were vacuum deposited through shadow masks. Current–voltage characteristics of the OFET devices were recorded at room temperature under ambient conditions by using a Keithley 4200-SCS semiconductor parameter analyzer (Tektronix, Beaverton, OR, USA). Field-effect hole mobilities of the OFET devices were extracted from the square root of the drain current (in the rage of *V*_g_ = −60 to −80 V for 4TDa and −40 to −70 V for 4TDb) in the saturation regime (*V*_d_ = −60 V) by using the following equation,

(1) $$\mu =\frac{2L}{WC_{i}}\cdot {\left(\frac{d\sqrt{\left|I_{d}\right|}}{dV_{g}}\right)}^{2}$$

where *C_i_* is the capacitance of the gate insulator, and *W* and *L* are the channel width and length, respectively. The average threshold voltage and hole mobility were obtained over more than 10 devices for both 4TDa and 4TDb.

### 2.3. Synthesis

The aromatic oligothiophene precursors for *n*TD, namely 4,4′-dihexyl-2,2′-bithiophene, 4,4″-dihexyl-2,2′:5′,2″-terthiophene, 4,4′′′-dihexyl-2,2′:5′,2″:5″,2′′′-quaterthiophene, and 3′,4,4″,4′′′-tetrahexyl-2,2′:5′,2″:5″,2′′′-quaterthiophene were synthesized according to the literatures [23,24].

### 2.3.1. 4,4′-Dihexyl-5*H*,5′*H*-[2,2′-bithiophenylidene]-5,5′-dione (2TD)

Under inert gas atmosphere, to a solution of 4,4′-dihexyl-2,2′-bithiophene (501 mg, 1.50 mmol) in THF (38 mL) was added n-butyllithium (1.57 M in hexane) (3.3 mL, 5.2 mmol) dropwise at 0 °C. After stirring for 1.5 h, triisopropyl borate (1.21 mL, 5.28 mmol) was added. After stirring at room temperature for 30 min, the reaction mixture was diluted with ammonium chloride aqueous solution (0.5 M, 20 mL), and the organic layer was separated. The organic layer was then washed with water (10 mL) three times. The organic layer was added to a solution of 30% hydrogen peroxide (30 mL) and ether (30 mL), and the reaction mixture was stirred vigorously for 15 min. Then, the organic layer was washed with water (100 mL) and brine (100 mL), after which it was dried over anhydrous sodium sulfate. Then, the mixture was passed through a short silica-gel column (ϕ4 cm, 5 cm long) six times. The solvent was evaporated under reduced pressure, and the crude product was purified by silica gel column chromatography (eluent: hexane/DCM = 4/1) followed by recrystallization from chloroform and hexane to give an isomer mixture of 2TD as a bright yellow solid (135 mg, 0.37 mmol, 25%). An analytically pure (*E*)-isomer sample for NMR was obtained by silica gel column under dark. Mp. 123–125 °C. ^1^H-NMR (CDCl_3_, 500 MHz): δ (ppm) (*E*-isomers) 7.43 (s, 2H), 2.47 (t, 4H, *J* = 7.2 Hz), 1.59 (quintet, 4H, *J* = 7.2 Hz), 1.31–1.37 (m, 12H), 0.89 (t, 6H, *J* = 7.0 Hz). ^13^C-NMR (CDCl_3_, 125 MHz): δ (ppm) (*E*-isomers) 191.98, 148.54, 140.07, 133.73, 31.45, 28.98, 28.10, 27.19, 22.49, 14.02. IR: ν (cm^−1^) 3041, 2954, 2933, 1681, 1664, 1601, 1467, 1178, 678, 530. HRMS (FD) *m/z* [M]^+^ Calcd for C_20_H_28_O_2_S_2_ 364.15307. Found: 364.15309. Anal. Calcd for C_20_H_28_O_2_S_2_: C, 65.89; H, 7.74. Found: C, 65.75; H, 7.66%.

### 2.3.2. 4,4″-Dihexyl-5*H*,5″*H*-[2,2′:5′,2″-terthiophene]-5,5″-dione (3TD)

In a similar manner to the synthesis of 2TD, the crude product of 3TD was obtained from 4,4″-dihexyl-2,2′:5′,2″-terthiophene (625 mg, 1.50 mmol) and purified by silica gel column chromatography (eluent: hexane/DCM = 3/1) followed by recrystallization from chloroform to give an isomer mixture of 3TD as a red solid (200 mg, 0.45 mmol, 30%). An analytically pure (*EE*)-isomer sample for NMR was obtained by silica gel column under dark. Mp. 181–182 °C. ^1^H-NMR (CDCl_3_, 500 MHz): *δ* (ppm) (*EE-isomer*) 7.56 (s, 2H), 7.32 (s, 2H), 2.44 (t, 2H, *J* = 7.7 Hz), 1.58 (quintet, 4H, *J* = 7.5 Hz), 1.39–1.29 (m, 12H), 0.89 (t, 6H, *J* = 7.0 Hz). ^13^C-NMR (CDCl_3_, 125 MHz): *δ* (ppm) (*EE*-isomer) 191.55, 143.84, 143.46, 137.57, 130.18, 129.81, 31.518, 29.02, 28.392, 27.063, 22.53, 14.06. IR: *ν* (cm^−1^) 2959, 2928, 2857, 1670, 1572, 1468, 1229, 1163, 783, 571. HRMS (FD) *m*/*z* [M]^+^ Calcd for C_24_H_30_O_2_S_3_ 446.14079. Found: 446.14059. Anal. Calcd for C_24_H_30_O_2_S_3_: C, 64.53; H, 6.77. Found: C, 64.70; H, 6.77%.

### 2.3.3. 4,4′′′-Dihexyl-5*H*,5′′′*H*-[2,2′:5′,2″:5″,2′′′-quaterthiophene]-5,5′′′-dione (4TDa)

In a similar manner to the synthesis of 2TD, the crude product of 4TDa was obtained from 4,4′′′-dihexyl-2,2′:5′,2″:5″,2′′′-quaterthiophene (374 mg, 0.75 mmol) and purified by recrystallization from chloroform followed by silica gel column chromatography (eluent: hexane/DCM = 1/3) to give an isomer mixture of 4TDa as a dark blue solid (28 mg, 0.05 mmol, 7%). Mp. 205–206 °C. ^1^H-NMR (CDCl_3_, 700 MHz): *δ* (ppm) (mixture of stereoisomers) 7.38, 7.53, 7.39, 7.30, 7.22, 7.04, 6.94, 2.48–2.43 (m), 1.61–1.58 (m), 1.37–1.28 (m), 0.91–0.88 (m). As a result of the possible six isomers having similar chemical shifts and coupling constants with each other, which were simulated by DFT methods, the assignment of the NMR signals to a specific isomers for estimating the isomer ratio was difficult. ^13^C-NMR (CDCl_3_, 175 MHz): *δ* (ppm) (mixture of stereoisomers) 192.08, 191.30, 191.25, 145.17, 142.78, 138.85, 138.83, 138.68, 137.37, 132.82, 131.69, 131.15, 131.05, 130.27, 128.22, 31.60, 29.08, 28.65, 27.20, 22.56, 14.01. IR: *ν* (cm^−1^) 2955, 2928, 2857, 1661, 1566, 1379, 1234, 1223, 1153, 1084, 876, 781, 669, 548. HRMS (FD) *m*/*z* [M]^+^ Calcd.: for C_28_H_32_O_2_S_4_ 528.12851. Found: 528.12873. Anal. Calcd for C_28_H_32_O_2_S_4_: C, 63.60; H, 6.09. Found: C, 63.55; H, 6.09%.

### 2.3.4. 3′,4,4″,4′′′-Tetrahexyl-5*H*,5′′′*H*-[2,2′:5′,2″:5″,2′′′-quaterthiophene]-5,5′′′-dione (4TDb)

In a similar manner to the synthesis of 2TD, the crude product of 4TDb was obtained from 3′,4,4″,4′′′-tetrahexyl-2,2′:5′,2″:5″,2′′′-quaterthiophene (334 mg, 0.50 mmol) and purified by silica gel column chromatography (eluent: hexane/DCM = 11/9) followed by recrystallization from chloroform to give an isomer mixture of 4TDb as a deep green solid (73 mg, 0.10 mmol, 21%). Mp. 172–173 °C. ^1^H-NMR (CDCl_3_, 700 MHz): *δ* (ppm) (mixture of stereoisomers) 7.58, 7.46, 7.08, 7.05, 6.99, 6.96, 6.87, 6.79, 2.73, 2.68, 2.43, 1.71, 1.65, 1.59, 1.58, 1.43, 1.32–1.36 (m), 0.90–0.92 (m). As a result of the possible six isomers having similar chemical shifts each other, which were simulated by DFT methods, assignment of the NMR signals to a specific isomers for estimating the isomer ratio was difficult. ^13^C-NMR (CDCl_3_, 175 MHz): *δ* (ppm) (mixture of stereoisomers) 193.24, 190.49, 190.07, 149.61, 149.46, 149.03, 148.10, 147.63, 147.30, 146.91, 145.57, 145.51, 144.11, 143.77, 143.63, 143.49, 143.25, 143.20, 143.01, 142.79, 142.59, 142.32, 142.15, 142.05, 141.89, 141.66, 141.24, 140.99, 140.64, 140.32, 138.90137.59, 137.19, 136.29, 135.38, 135.33, 134.90, 134.75, 134.41, 133.90, 133.74, 133.54, 133.43, 133.27, 133.12, 132.19, 132.05, 131.90, 131.59, 128.38, 127.76, 127.60, 127.14, 125.96, 124.80, 124.41, 124.04, 114.60, 114.40, 31.95, 31.62, 31.55, 31.07, 31.00, 30.66, 30.64, 30.63, 30.11, 30.08, 29.73, 29.66, 29.51, 29.37, 29.18, 29.05, 29.00, 28.84, 28.71, 28.58, 28.52, 28.37, 27.91, 27.16, 27.08, 26.89, 26.23, 23.91, 22.55, 22.51, 22.38, 22.30, 13.94, 13.80, 13.75. IR: ν (cm^−1^) 2955, 2924, 2855, 1645, 1564, 1489, 1157, 669, 550. (EI) *m*/*z* = (Not observed) [M^+^]. HRMS (MALDI) *m*/*z* [M]^+^ Calcd: for C_40_H_56_O_2_S_4_ 696.31577. Found: 696.31569. Anal. Calcd for C_40_H_56_O_2_S_4_: C, 68.92; H, 8.10. Found: C, 68.60; H, 7.93%.

## 3. Results

### 3.1. Theoretical Calculations

The critical requirement for p-type organic semiconductors is a high-lying HOMO energy level (*E*_HOMO_) that ensures hole injection from the electrode and a small reorganization energy for hole transport (λ_hole_). In order to evaluate the potential of the carbonyl-terminated quinoidal oligothiophenes (*n*TDs) as p-type organic semiconductors, we theoretically investigated the electronic structures of *n*TDs by DFT calculations on the model compounds for 2TD, 3TD, 4TDa, and 4TDb, where the n-hexyl groups are substituted with methyl groups. *n*TDs can have stereoisomers (*E/Z*-isomers) owing to the existence of inter-ring double-bond(s), and all the possible isomers were calculated. However, differences between isomers are marginal (see Table S1 for detail), and the values tabulated in Table 1 are the averaged ones for *E*_HOMO_, LUMO energy levels (*E*_LUMO_), *E*_g_, and *λ*_hole_ and reorganization energy for electron transport (*λ_e_*_lectron_) calculated at the (U)B3LYP/6-311G** level [21]. Corresponding aromatic oligothiophenes (*n*Ts), i.e., bithiophene (2T), terthiophene (3T), and quaterthiophene (4T), were also calculated to compare the electronic structures.

The extension of π-conjugation in the quinoidal thiophenes (*n*TD, *n* = 2~4) effectively elevates the *E*_HOMO_ from −6.41 (2TD) to −5.30 eV (4TD), which is more pronounced than that in the aromatic oligothiophene series (*n*T, *n* = 2~4). On the other hand, the *E*_LUMO_ of *n*TDs is less sensitive to the π -extension than the *E*_HOMO_ and is always calculated to be around −3.3 to −3.5 eV, which is again contrasted to *n*Ts, where the π -extension effectively lowers the *E*_LUMO_. As a result, 4TD, the most π-extended quinoidal thiophene in this study, has almost comparable *E*_HOMO_ with 4T, which was reported to behave as a p-type organic semiconductor [25,26]. It should be also mentioned that no open-shell character in the grand state was confirmed even for the largest 4TD in the spin-unrestricted calculations with the broken symmetry conditions, indicating that the carbonyl terminal groups cannot stabilize the diradical ground state, which is contrasted to the dicyanoemethylene terminal group [27,28].

The *λ*_hole_s of the *n*TDs are almost half of *λ*_electron_s (Table 1), indicating that the *n*TDs are inherently suitable for effective hole transport. Furthermore, the *λ*_hole_s of the *n*TDs are markedly smaller than those of the corresponding *n*Ts; for example, *λ*_hole_s of 4TDs are calculated to be approximately 0.19 eV, whereas that of 4T is approximately 0.3 eV, indicating that 4TDs likely have a certain potential as p-type organic semiconductors.

**Figure 2:**
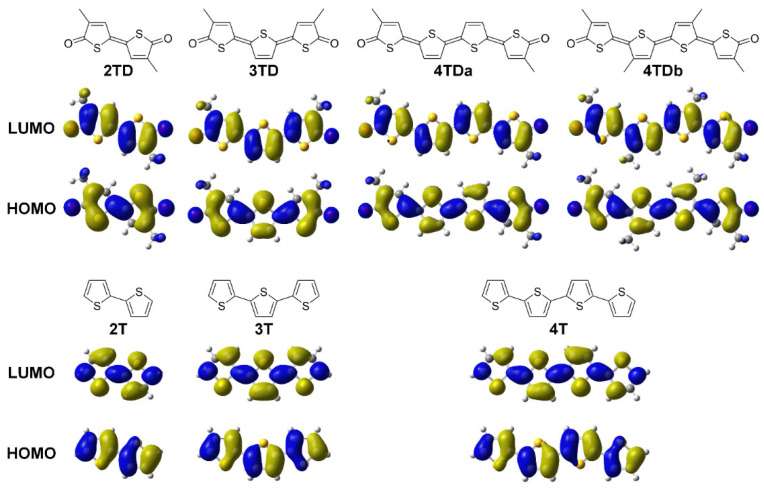
HOMOs and LUMOs of (E)-2TD, (EE)-3TD, (EEE)-4TDa, (EEE)-4TDb, 2T, 3T, and 4T.

Another important piece of information that we can obtain from theoretical calculations is the spatial distribution of HOMO and LUMO on the molecular framework. Figure 2 shows the HOMOs and LUMOs of the (*E*)-isomers of *n*TDs and their aromatic counterparts. It is worth mentioning that the spatial distribution of HOMO and LUMO in *n*TDs are opposite to those of *n*Ts as a consequence of the altered bond alternation. Importantly, the 3p-orbitals of the sulfur atoms in nTDs largely contribute to the HOMOs, whereas the nodal planes are on the sulfur atoms in *n*Ts’ HOMOs. The large contribution of the sulfur atoms to the HOMO of *n*TDs should be beneficial for the effective intermolecular overlap of HOMO in the solid state, which is favorable for effective hole transport [29].

From the theoretical calculations, the characteristic electronic features of *n*TDs can be summarized as high-lying *E*_HOMO_s, small *λ*_hole_s, and a large contribution of sulfur atoms to HOMOs, all of which are desirable for p-type organic semiconductors. Thus, we can rationally conclude that *n*TDs can be potential candidates for p-type organic semiconductors.

### 3.2. Synthesis and Chatacterization

**Scheme 1:**
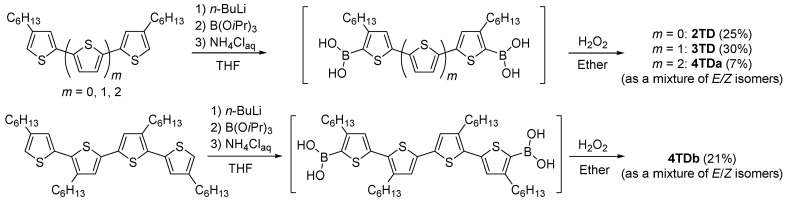
Synthesis of *n*TDs.

The quinoidal oligothiophenes, 2TD, 3TD, 4TDa, and 4TDb were synthesized from the corresponding oligothiophenes by a one-pot reaction previously reported for the synthesis of quinoidal acenedithiophenediones (Scheme 1) [30,31]. For the synthesis of 2TD, 4,4′-dihexylbithiophene was lithiated at the α-positions with *n*-butyllithium and borylated with triisopropyl borate, and the resulting diboronic ester was hydrolyzed with an aqueous ammonium chloride solution to in situ generate 2T-diboronic acid (Scheme 1). Then, diboronic acid was subjected to an oxidative rearrangement reaction with hydrogen peroxide followed by a hydrolysis and tautomerization, and finally a dehydrogenative oxidation gave 2TD as a mixture of the (*E*)- and (*Z*)-isomers in a 25% isolated yield (see also Scheme S1). In a similar manner, 3TD, 4TDa, and 4TDb were synthesized from the corresponding oligothiophene precursors in yields of 30%, 7%, and 21%, respectively. Although the isolated yields of the quinoidal oligothiophenes are not high, they would be reasonable for such a complicated multi-step conversion (Scheme S1). All the quinoidal oligothiophenes were obtained as a mixture of the isomers and were well identified by NMR (Figures S1–S8, HRMS (Figures S9–S12), and elemental analysis. In addition, IR spectroscopy demonstrated characteristic stretching vibrations assignable to the carbonyl terminal group (2TD: 1681 cm^−1^, 3TD: 1670 cm^−1^, 4TDa: 1661 cm^−1^, 4TDb: 1645 cm^−1^). Two-dimensional thin-layer chromatography (2D-TLC) analysis of the compounds revealed that the (*E*/*Z*) and (*EE*/*EZ*/*ZZ*) stereoisomers of 2TD and 3TD, respectively, have different R_f_ values, thus being separable by silica gel column chromatography (Figure S13a,b). However, the non-diagonal spots in the analyses for 2TD and 3TD indicate that the stereoisomers in 2TD and 3TD are interconvertible to each other on a thin-layer chromatography (TLC) under ambient conditions, in which the isomerization is assisted by the irradiation of room light. Therefore, by carrying out silica gel column chromatography under dark, some of the stereoisomers of 2TD and 3TD were successfully isolated though the isolated pure isomers, which were easily isomerized with the exposure to room light to become isomer mixtures again. On the other hand, 4TDa and 4TDb showed only one spot in the 2D-TLC, which indicates that the R_f_ values of the isomers are too similar to each other and/or isomerization occurs too quickly on a TLC plate during the analysis (Figure S13c,d).

**Figure 3:**
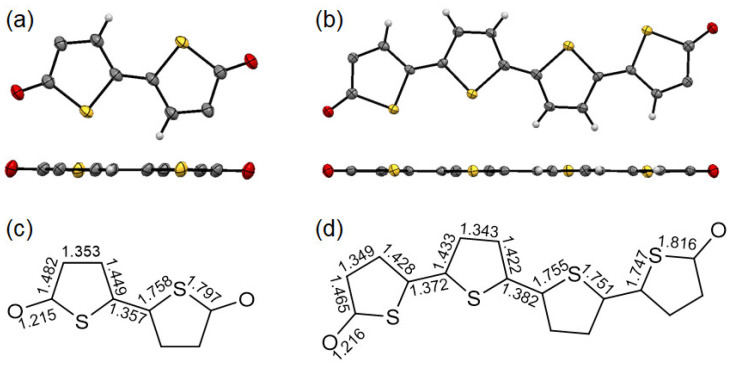
Molecular structures and the bond lengths (Å) of (*E*)-2TD (**a**,**c**) and (*ZEZ*)-4TDa (**b,d**) elucidated by single-crystal X-ray analyses. The alkyl groups are omitted for clarity.

Although the spectroscopic characterization of the quinoidal oligothiophenes was decently done, the existence of isomers made the characterization of the quinoidal oligothiophenes ambiguous. Thus, we tried to carry out single-crystal X-ray analysis, and the structures of 2TD and 4TDa were successfully elucidated (Table S2). Figure 3a,b show the π-conjugated core part of 2TD with the (*E*)-form and 4TDa with the (*ZEZ*)-form. Both molecules have highly planar π-core structures with the maximum deviation of 0.024 and 0.023 Å, respectively. The bond lengths in the π-conjugated cores clearly show the quinoidal bond-length alternation; the terminal C-O bonds, the C-C bonds at the thiophene c-sides, and the inter-ring C-C bonds have the double bond nature, whereas the C-C bonds of the thiophene b-sides have the single bond nature.

### 3.3. Physicochemical Properties

**Figure 4:**
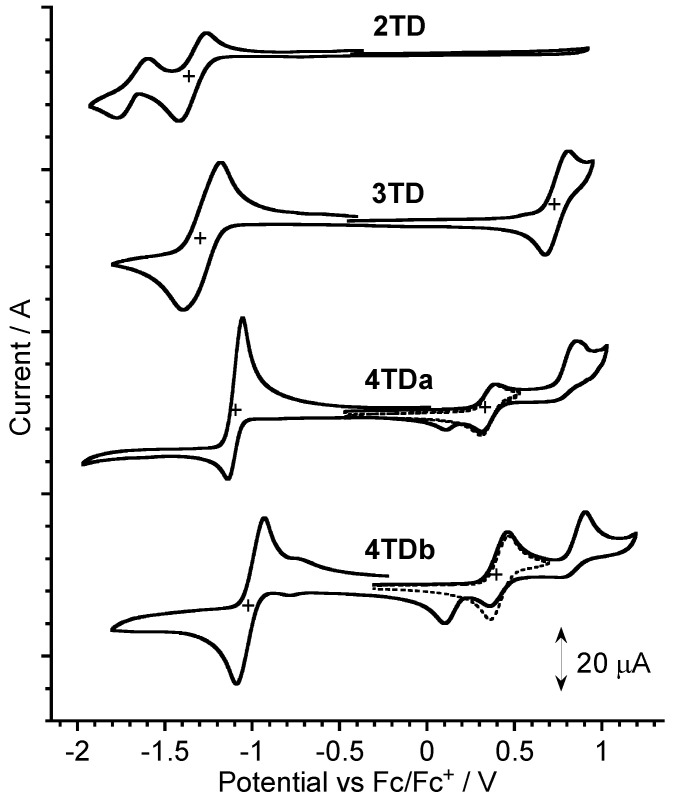
Cyclic voltammograms of *n*TDs. Dotted voltammograms were recorded by reversing the scanning direction after the first oxidation.

*E*_HOMO_s and *E*_LUMO_s of *n*TDs were electrochemically estimated by cyclic voltammetry (Figure 4). 3TD, 4TDa, and 4TDb showed (quasi)reversible one- or two-electron oxidation and reduction current responses, whereas 2TD showed only a reversible reduction current response, indicating that the oxidation potential seems to be out of the potential window in the present electrochemical conditions (−3.3 to −5.9 eV below the vacuum level), which is consistent with the theoretically estimated *E*_HOMO_ (Table 1). The first oxidation and reduction half-wave potentials of the compounds are summarized in Table 2. The *E*_HOMO_s and *E*_LUMO_s of *n*TDs were estimated under the assumption that the half-wave redox potential of ferrocene/ferrocenium (Fc/Fc^+^) is 4.8 eV below the vacuum level [17]. The *E*_LUMO_s of the compounds are ranging from −3.5 to −3.7 eV, which are much higher than that of a previously reported quinoidal terthiophene terminated with dicyanomethylene groups (*E*_LUMO_ = −4.2 eV) [13]. This is consistent with the fact that the carbonyl terminal group is a weaker electron-withdrawing group than the dicyanomethylene group, and the carbonyl termination is an appropriate strategy to suppress the decrease of *E*_LUMO_ of thienoquinodal compounds. On the other hand, the *E*_HOMO_s of *n*TDs markedly increase from below −5.9 to −5.1 eV with the π-extension, which is again consistent with the theoretical calculations.

**Figure 5:**
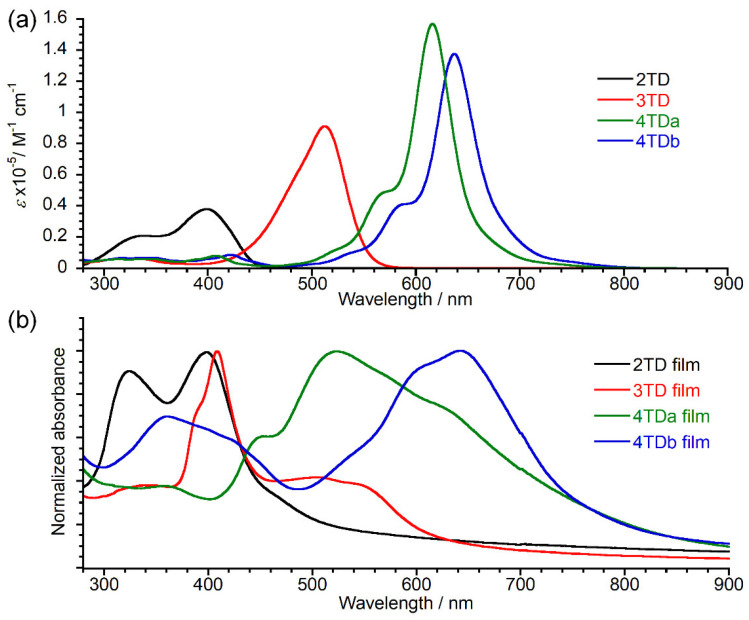
UV-vis absorption of *n*TDs in chloroform solution (**a**) and in thin film (**b**).

Figure 5 shows the absorption spectra of *n*TDs both in the chloroform solution and the thin film state. Extension of the π-conjugation system induces both bathochromic and hyperchromic shifts in the lowest-energy absorption band assigned to the HOMO–LUMO (π–π*) transition. The absorption onsets of 2TD, 3TD, 4TDa, and 4TDb in the chloroform solution are 398, 513, 616, and 637 nm, respectively, which correspond to the optical energy gaps (*E*_g_) of 2.8, 2.2, 1.8, and 1.7 eV, respectively (Table 3). These values are in good agreement with those estimated from electrochemistry (Table 2).

The thin films deposited by spin-coating show rather broader absorption bands. Various intermolecular interaction between the stereoisomers might be the reason for the broad and complicated spectral shape compared to those in solution. In the case of thin films of 3TD, significant blue-shifts of 105 nm (3TD) were observed. Similar blue-shifts were previously reported for related thienoquinoidal compounds, where formation of the H-aggregate in the thin-film state is attributed to the shift [32]. The ionization potentials (IPs) at the thin-film state were also measured by photoelectron spectroscopy in air (Table 3, Figure S14). The IPs of 4TDa and 4TDb are 5.3 and 5.1 eV, respectively, which are in good agreement with the electrochemically estimated *E*_HOMO_s. In contrast, the IP of 3TD (5.8 eV) is much larger than that estimated from the electrochemical *E*_HOMO_ (5.5 eV). Although the difference cannot be clearly explained, the molecular aggregation in the thin-film state that causes the large blue shift in the 3TD-thin film might be related.

### 3.4. Electronic Structure in the Solid State

As already mentioned in the section of theoretical calculations, *λ*_hole_ is an important molecular factor that is closely related to the hole transport properties. Another critical factor that governs the hole transport in the solid state is intermolecular electronic coupling (orbital overlap or transfer integral between HOMOs). Thus, it is critically important to have a decent packing structure for efficient hole transport. In this sense, the crystal structures of 2TD and 4TDa can provide insight into their solid-state electronic structures.

**Figure 6:**
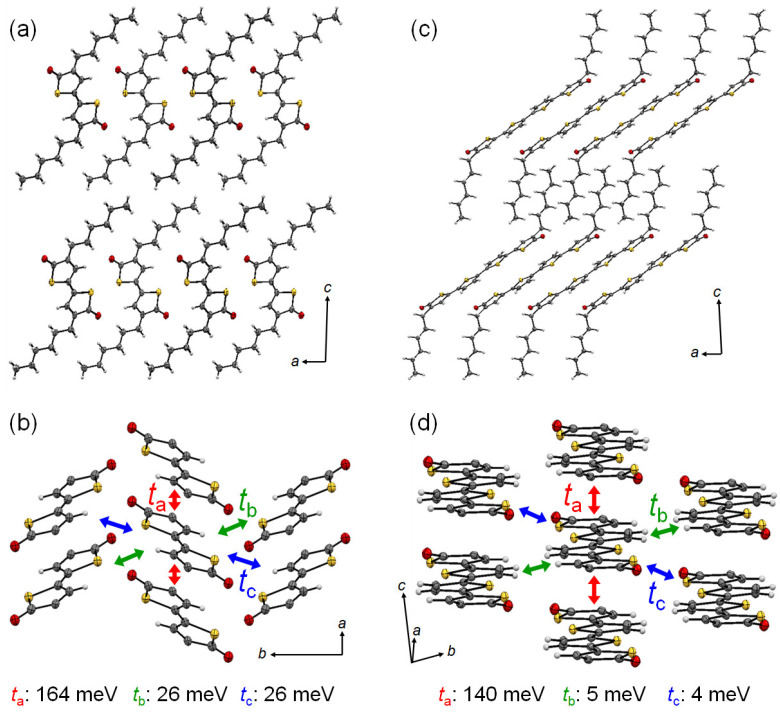
Packing structure and transfer integrals for hole transport of (*E*)-2TD (**a**,**b**) and (*ZEZ*)-4TDa (**c**,**d**). Alkyl groups were omitted for clarity in (**b**,**d**).

Both the packing structure of 2TD and 4TDa are classified into lamellar structures, in which the π-core part and the alkyl-chain layers are alternately stacked along the crystallographic *c*-axis direction (Figure 6a,c, respectively). Such lamellar structures have been commonly observed in the packing structures of organic semiconductors consisting of a rod-shaped π-core and long alkyl side groups; e.g., αω*-*dialkylated oligothiophenes [25]. In the π-core layers, both 2TD and 4TDa form π-stacking columnar structures with short π-stacking distances of 3.48 and 3.53 Å for 2TD and 4TDa, respectively, along the crystallographic *a*-axis direction (Figure 6b,d). Such short π-stacking distances are favorable for large intermolecular electronic couplings, and in fact, the transfer integrals of HOMOs (*t*_a_s) in the π-stacking columnar structure are calculated to be 164 and 140 meV for 2TD and 4TDa, respectively [22] (see Figure S15 for the transfer integrals for LUMOs), which are quite large among the already reported organic semiconductors [29]. In addition, moderately large electronic couplings (27 meV, Figure 6b) are confirmed in the inter-column direction in the 2TD crystal, indicating that 2TD can have a quasi-two-dimensional (2D) electronic structure for hole transport. On the other hand, the electronic coupling in the inter-columnar direction is very small in the packing structure of 4TDa (5 or 4 meV, Figure 6d), resulting in one-dimensional electronic structure. From these calculations, we can conclude that the quinoidal oligothiophene framework is an interactive structure that can afford very efficient intermolecular HOMO overlap, although the electronic structure of the present cases are rather low dimensional.

### 3.5. Fabrication and Evaluation of Thin Film Transistors

**Figure 7:**
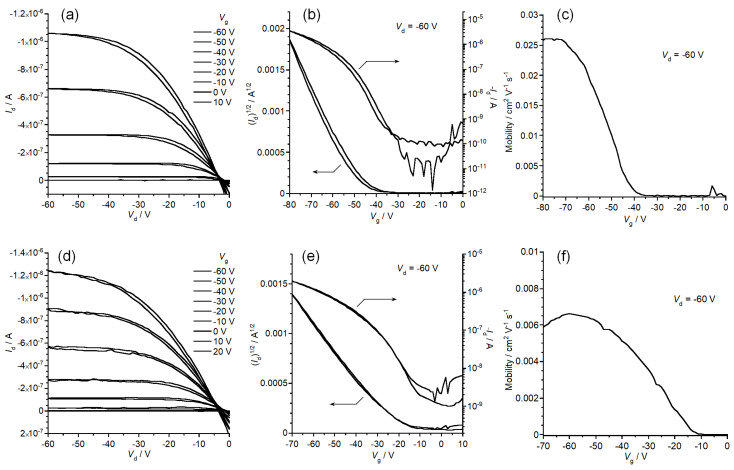
Output and transfer curves, gate-voltage-dependent mobilities of 4TDa (**a**–**c**) and 4TDb (**d**–**f**), respectively.

Bottom-gate-top-contact OFET devices consisting of spin-coated thin films of *n*TDs as the active layer and gold source and drain electrodes were fabricated on the Si/SiO_2_ substrate and evaluated in air under both the p- and n-channel operation conditions. The 2TD- and 3TD-based devices did not show any transistor response, which was probably because of problematic hole injection owing to their large IPs (*vide supra*). On the other hand, the OFETs with the 4TDa- and 4TDb-thin-films showed unipolar p-type FET behaviors (Figure 7). The hole mobilities of the 4TDa- and 4TDb-based devices extracted from the saturation regime are 0.026 and 0.0056 cm^2^ V^−1^ s^−1^, respectively. Considering the fact that the present 4TDs thin films likely consist of several stereoisomers, which cannot be suitable for effective carrier transport in the solid state, we could say that the small reorganization energies and capability for effective orbital overlap in the solid state of 4TDs can compensate for the drawback.

**Figure 8:**
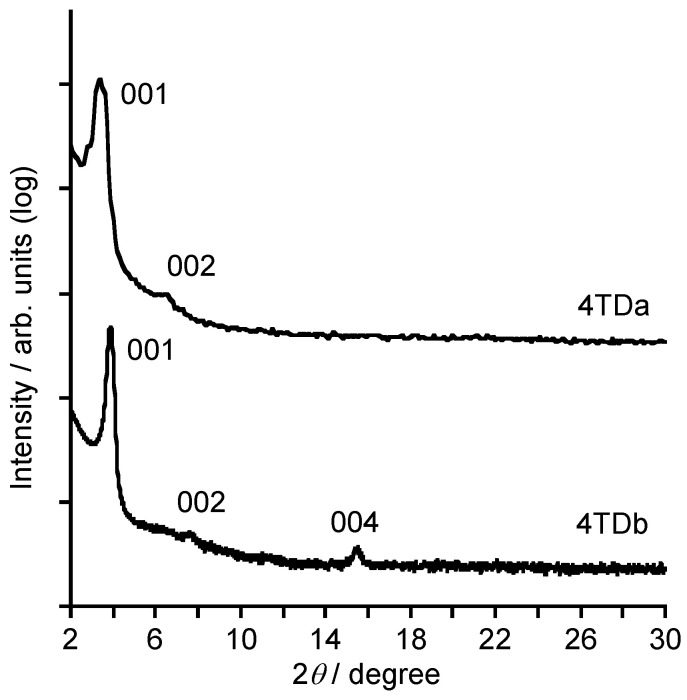
Out-of-plane X-ray diffraction patterns of spin-coated films of 4TDa and 4TDb.

Thin-film microstructural analyses of 4TDa and 4TDb thin films were carried out by X-ray diffraction (XRD) and atomic force microscopy (AFM) (Figure S16). Figure 8 shows the XRD patterns of 4TDa- and 4TDb-thin films on the Si/SO_2_ substrate. The appearance of clear peaks assignable to the 00*l* diffractions in both films indicates the formation of crystalline domains, but the crystallinity may not be very high, as the peaks are broad and weak in intensity. The *d*-spacing extracted from the 001 peak in the 4TDa thin-film is 26 Å, which is much longer than the crystallographic *c*-axis (17.5127(4) Å) of the single-crystal structure. This means that the packing structure in the thin-film state is significantly different from that in the single crystal, and this can be explained by the presence of stereoisomers in the thin film. Thus, it is not straightforward to correlate the carrier mobility in the devices and the packing structure in the thin film state. However, the packing structure of 4TDa in the single crystal cannot be beneficial for efficient carrier transport, provided that the crystallographic *ab*-plane is the conductive plane, because of the large inclination of the 4TDa core part against the *c*-axis (Figure 6c). In this regard, the *d*-spacing of 26 Å in the thin-film state is very promising for the formation of an efficient carrier transport path, as the 4TDa core part stands in a nearly upright manner, creating a thicker conduction layer in the lamella structure. It could be slightly different, but basically a similar packing structure can be postulated in the 4TDb thin film with *d*-spacing of 22.8 Å.

## 4. Conclusions

Prompted by the idea that quinoidal oligothiophenes, which have been long recognized as n-type or ambipolar organic semiconductors, could be a potential platform as p-type organic semiconductors, we have carried out the theoretical and experimental approach to p-type quinoidal oligothiophenes. The theoretical calculations revealed that the quinoidal oligothiophene framework is indeed a suitable one for a p-type organic semiconductor, a small reorganization energy for hole transport, and a large contribution of sulfur atoms to HOMO. Thus, we designed and synthesized a series of quinoidal oligothiophenes terminated with carbonyl groups, i.e., 2TD, 3TD, 4TDa, and 4TDb. The physicochemical evaluations of the series both at the molecular and solid-state level clearly endorsed the theoretical predictions. Furthermore, the solution-processed thin-film transistors based on 4TDa and 4TDb showed decent unipolar p-type transistor behaviors with hole mobilities of up to 0.026 cm^2^ V^−1^ s^−1^. The mobility itself is not comparable to the state-of-the-art materials, but we believe that it is important to experimentally confirm the rational ideas for new material design strategies. We hope that the present work would further promote new applications of quinoidal oligothiophenes by tuning the chemical structure as well as physical properties.

## Figures and Tables

**Table 1:** Highest occupied molecular orbital (HOMO) and lowest unoccupied molecular orbital (LUMO) energy levels (*E*_HOMO_ and *E*_LUMO_), energy gaps (*E*_g_), and reorganization energies for hole (*λ*_hole_) and electron (*λ*_electron_) transport of the model compound for carbonyl-terminated quinoidal oligothiophenes (*n*TDs) and the aromatic oligothiophene series (*n*Ts) calculated by the DFT method at the UB3LYP/6-311G** level. 2T: bithiophene, 2TD: 4,4′-dihexyl-5*H*,5′*H*-[2,2′-bithiophenylidene]-5,5′-dione, 3T: terthiophene, 3TD: 4,4″-dihexyl-5*H*,5″*H*-[2,2′:5′,2″-terthiophene]-5,5″-dione, 4T: quaterthiophene, 4TDa: 4,4′′′-dihexyl-, and 4TDb: 3′,4,4″,4′′′-tetrahexyl-dihexyl-5*H*,5′′′*H*-[2,2′:5′,2″:5″,2′′′-quaterthiophene]-5,5′′′-dione.

Cmpd	*E*_HOMO_ (eV)	*E*_LUMO_ (eV)	*E*_g_ (eV)	*λ*_hole_ (eV)	*λ*_electron_ (eV)
2TD	−6.41	−3.30	3.10	0.310	0.538
3TD	−5.71	−3.44	2.26	0.224	0.446
4TDa	−5.30	−3.53	1.77	0.190	0.388
4TDb	−5.19	−3.45	1.73	0.184	0.381
2T	−5.73	−1.51	4.22	0.376	0.334
3T	−5.38	−1.94	3.44	0.332	0.286
4T	−5.20	−2.17	3.03	0.303	0.259

**Table 2:** Oxidation and reduction potentials and estimated *E*_HOMO_s, *E*_LUMO_s, and *E*_g_s of *n*TDs.

Cmpd	*E*_ox_^1/2^ (V)	*E*_red_^1/2^ (V)	*E*_HOMO_^CV *a*^ (eV)	*E*_LUMO_^CV *b*^ (eV)	*E*_g_*^c^* (eV)
2TD	>1.1	−1.36	<−5.9	−3.44	>2.4
3TD	0.73	−1.30	−5.53	−3.50	2.03
4TDa	0.33	−1.10	−5.13	−3.70	1.43
4TDb	0.39	1.02	−5.19	−3.75	1.44

*^a^ E*_HOMO_ = −4.80 eV-*E*_ox_^1/2^. *^b^ E*_LUMO_ = −4.80 e-V*E*_red_^1/2^.*^c^ E*_g_ = *E*_LUMO_-*E*_HOMO_.

**Table 3:** The absorption maximum (*λ*_max_) and onset (*λ*_max_) wavelength, absorption coefficient (*ε*), optical energy gap (*E*_g_^opt^), and ionization potential (IP) of the *n*TDs in solution and in thin-film state.

Cmpd	In Solution				Film			
	λmax (nm)	λonset (nm)	ε (Lcm^−1^mol^−1^)	Egopt (eV)	λmax (nm)	λonset (nm)	Egopt (eV)	IP (eV)
2TD	398	445	3.9×10^4^	2.79	397	451	2.75	>6.2
3TD	513	556	9.2×10^4^	2.23	408	620	2.00	5.8
4TDa	616	704	1.6×10^5^	1.76	523	756	1.64	5.3
4TDb	637	724	1.4×10^5^	1.71	642	760	1.63	5.1

## Supplementary Materials

The following are available online at https://www.mdpi.com/1996-1944/13/13/3020/s1, Table S1: *E*_HOMO_ and *E*_LUMO_, *E*_g_, *λ*_hole_, and *λ*_electron_ of all *E/Z* isomers of the model compounds for *n*TDs and *n*Ts, Scheme S1: Details of the multi-step conversion in the one-pot synthesis of *n*TDs from the corresponding *n*T-precursors, Figure S1: ^1^H NMR spectra of 2TD, Figure S2: ^13^C NMR spectra of 2TD, Figure S3: ^1^H NMR spectra of 3TD, Figure S4: ^13^C NMR spectra of 3TD, Figure S5: ^1^H NMR spectra of 4TDa, Figure S6: ^13^C NMR spectra of 4TDa, Figure S7: ^1^H NMR spectra of 4TDa, Figure S8: ^13^C NMR spectra of 4TDb, Figure S9: HRMS spectrum of 2TD, Figure S10: HRMS spectrum of 3TD, Figure S11 HRMS spectrum of 4TDa, Figure S12: HRMS spectrum of 4TDb, Figure S13: 2D-TLC analyses, Figure S14: Photoemission yield spectra of thin films of 2TD, 3TD, 4TDa, and 4TDb, Table S2: crystallographic data for (*E*)-2TD and (*ZEZ*)-4TDa, Figure S15: Packing structure and transfer integrals for electron transfer of (*E*)-2TD and (*ZEZ*)-4TDa, Figure S16: AFM images of 4TDa and 4TDb thin films.
