# Supplementary material for: Carbonyl-Terminated Quinoidal Oligothiophenes as p-Type Organic Semiconductors
Source: Materials (Basel). 2020 Jul 6;13(13):3020. doi: 10.3390/ma13133020 (PMC7372439; doi:10.3390/ma13133020)
Supplement: Supplementary file 1 [file materials-13-03020-s001.pdf]

materials

MDPI

Supplementary Materials: Carbonyl-Terminated Quinoidal Oligothiophenes for p-Type Organic Semiconductors

Takato Asoh, Kohsuke Kawabata and Kazuo Takimiya *

Table S1. HOMO and LUMO energy levels (E_{HOMO} and E_{LUMO}), energy gaps (E_g), and reorganization energies for hole ($_{hole}$) and electron ($_{electron}$) transfer of all the *E*/*Z* isomers of the model compounds for *n*TDs and *n*Ts calculated by DFT method at the UB3LYP/6-311G** level.

cmpd	E/Z isomers	E_{HOMO} (eV)	E_{LUMO} (eV)	E_g (eV)	$_{hole}$ (eV)	$_{electron}$ (eV)
2TD	E	−6.40	−3.33	3.08	0.332	0.540
	Z	−6.41	−3.28	3.13	0.289	0.536
	average	−6.41	−3.30	3.10	0.310	0.538
3TD	EE	−5.71	−3.49	2.22	0.232	0.448
	EZ	−5.71	−3.44	2.26	0.223	0.446
	ZZ	−5.70	−3.40	2.30	0.217	0.445
	average	−5.71	−3.44	2.26	0.224	0.446
4TDa	EEE	−5.31	−3.62	1.69	0.198	0.389
	EEZ	−5.30	−3.58	1.72	0.194	0.387
	EZE	−5.32	−3.57	1.75	0.192	0.392
	EZZ	−5.30	−3.53	1.77	0.190	0.388
	ZEZ	−5.30	−3.55	1.75	0.191	0.385
	ZZZ	−5.28	−3.49	1.79	0.186	0.384
	average	−5.30	−3.56	1.74	0.192	0.388
4TDb	EEE	−5.17	−3.47	1.70	0.197	0.390
	EEZ	−5.19	−3.48	1.71	0.187	0.380
	EZE	−5.18	−3.43	1.75	0.192	0.393
	EZZ	−5.19	−3.43	1.76	0.182	0.381
	ZEZ	−5.21	−3.48	1.73	0.176	0.371
	ZZZ	−5.20	−3.43	1.77	0.172	0.369
	average	−5.19	−3.45	1.73	0.184	0.381

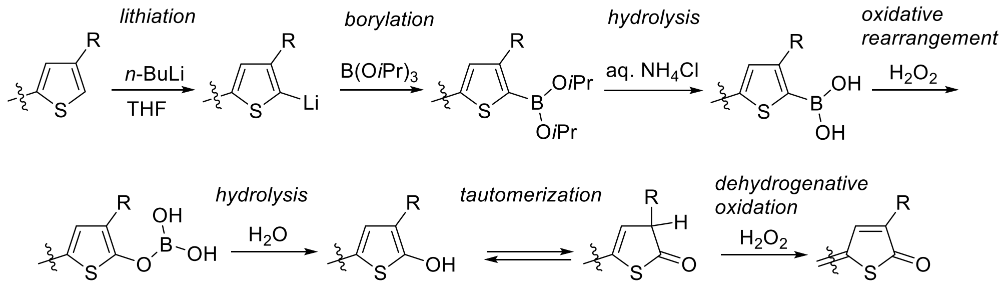

Scheme S1. Details of the multi-step conversion in the one-pot synthesis of *n*TDs from the corresponding *n*T-precursors.

^{1}H and ^{13}C NMR spectra

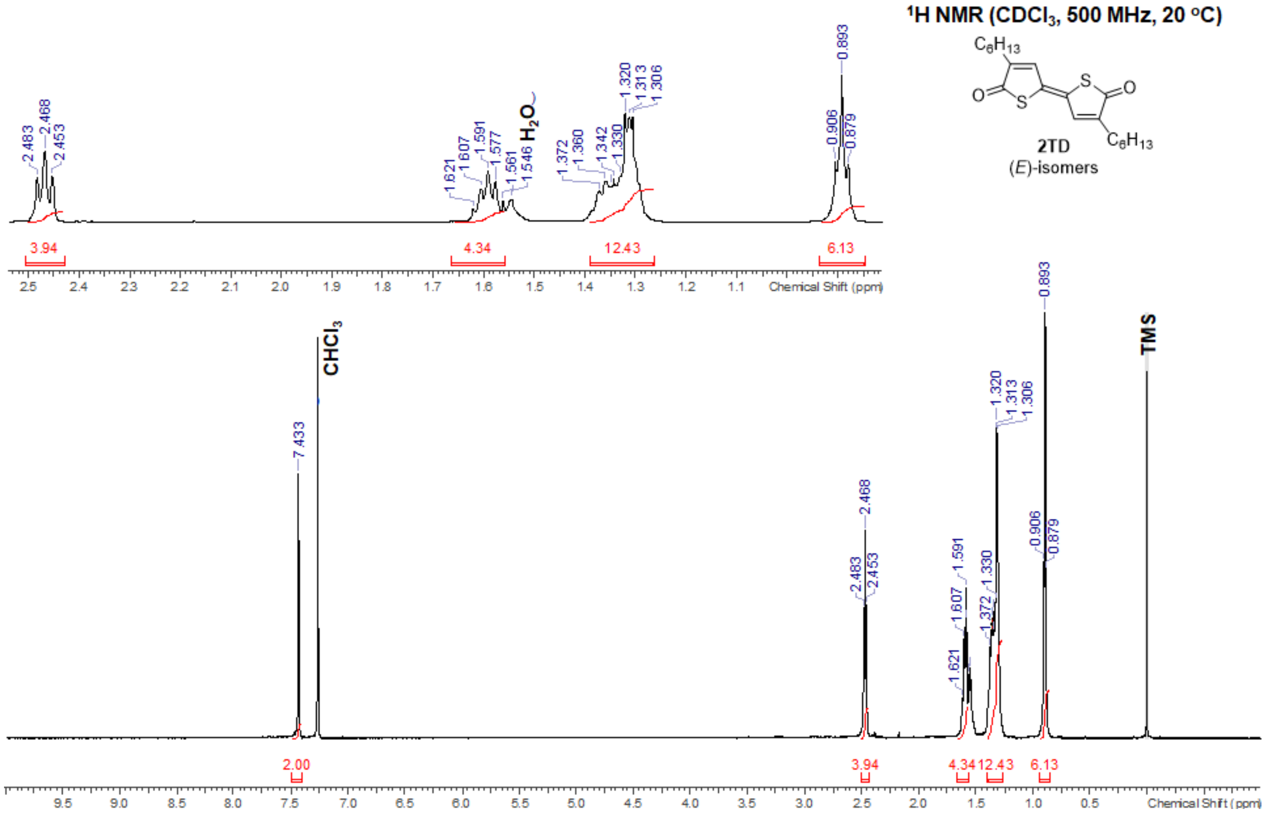

Figure S1. ^{1}H NMR spectra of (*E*)-2TD.

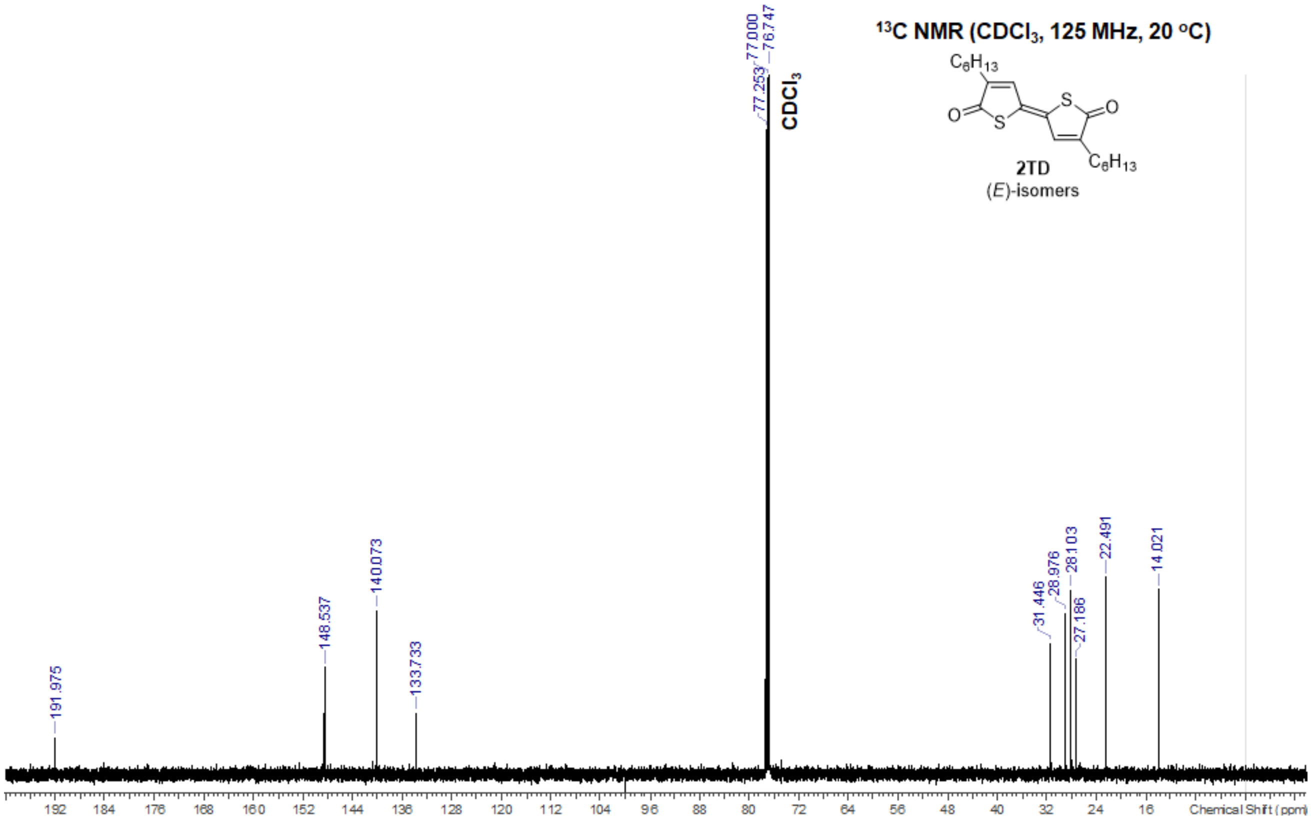

Figure S2. ^{13}C NMR spectra of (*E*)-2TD.

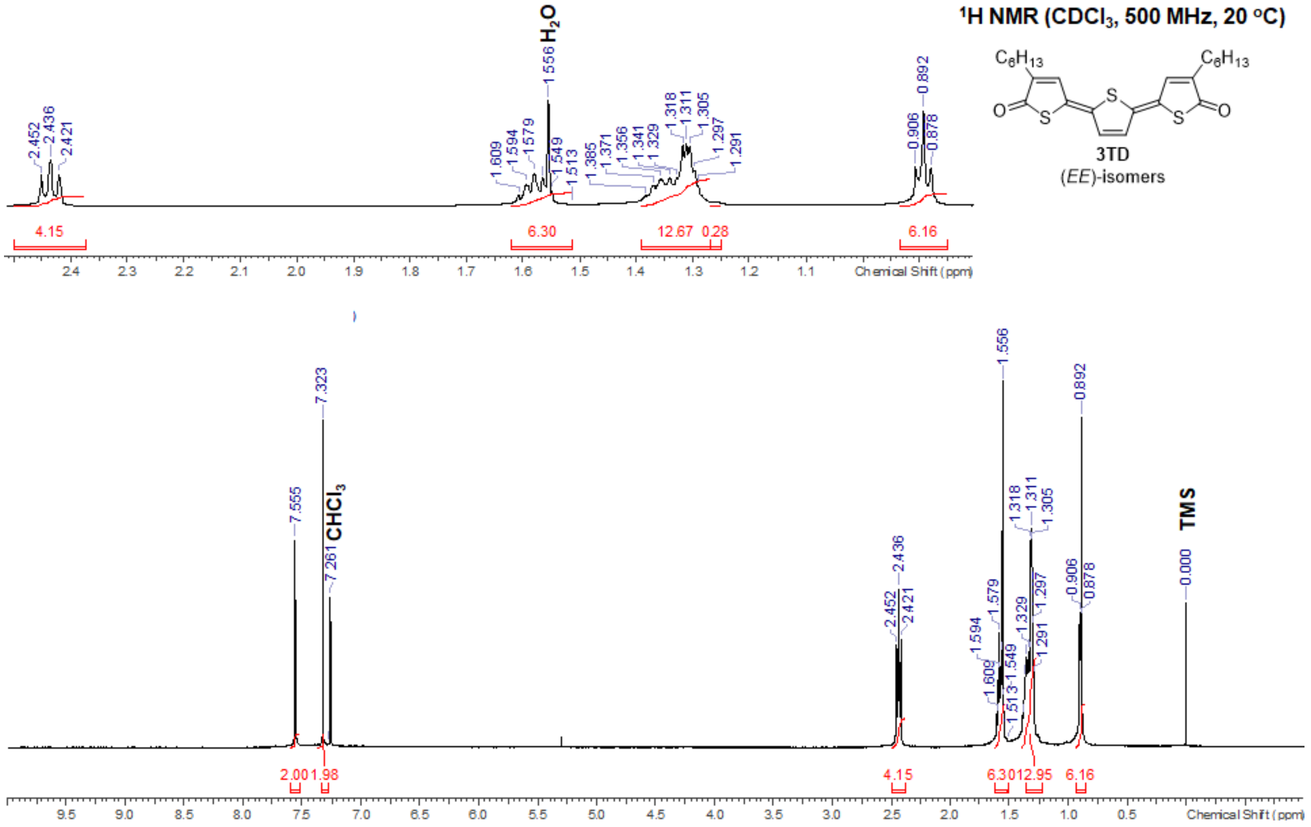

Figure S3. ^{1}H NMR spectra of (*EE*)-3TD.

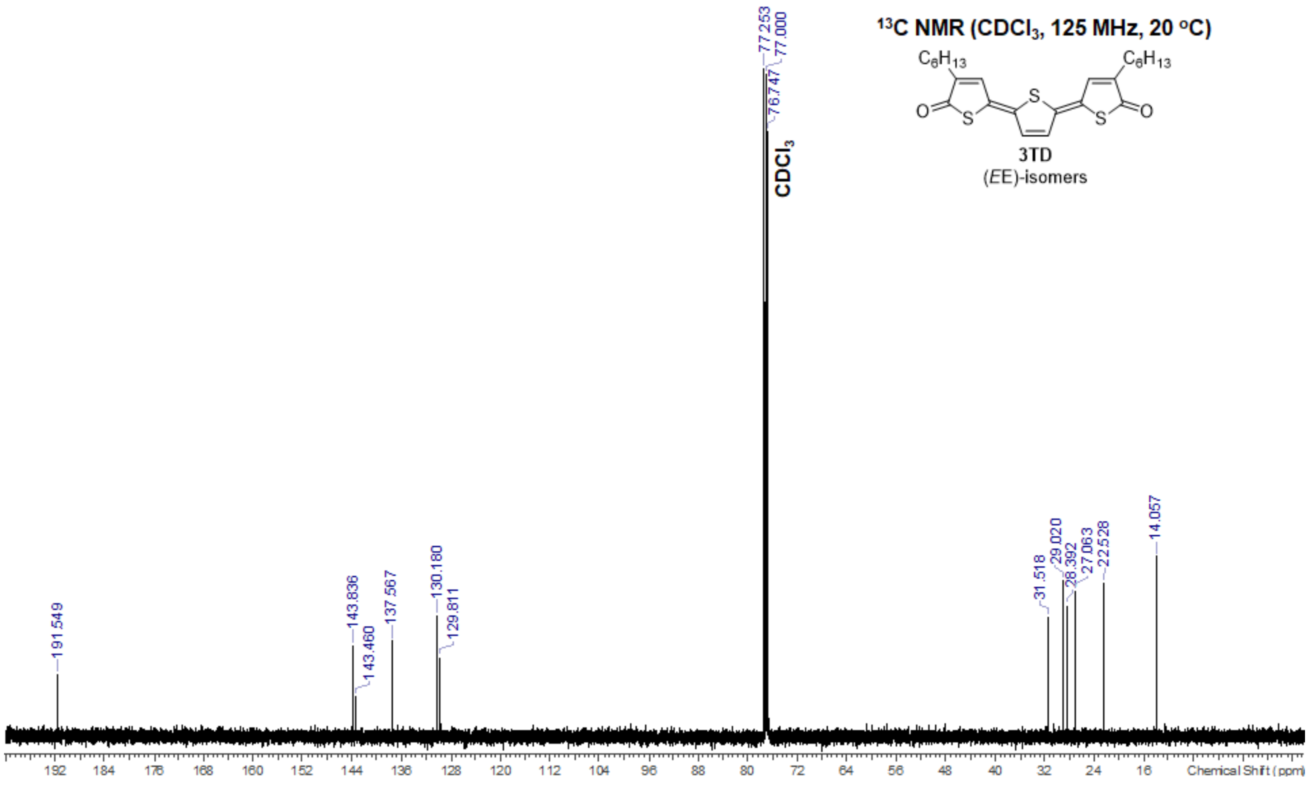

Figure S4. ^{13}C NMR spectra of (*EE*)-3TD.

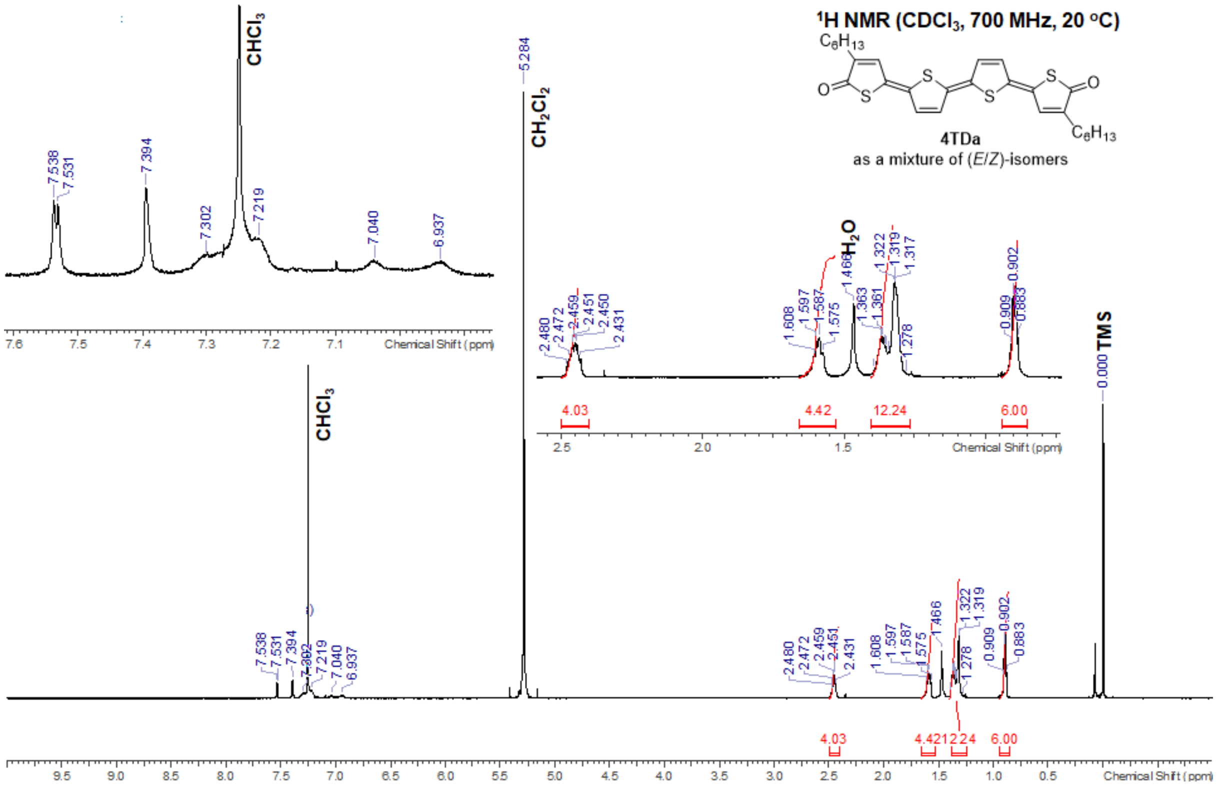

Figure S5. ^{1}H NMR spectra of 4TDa.

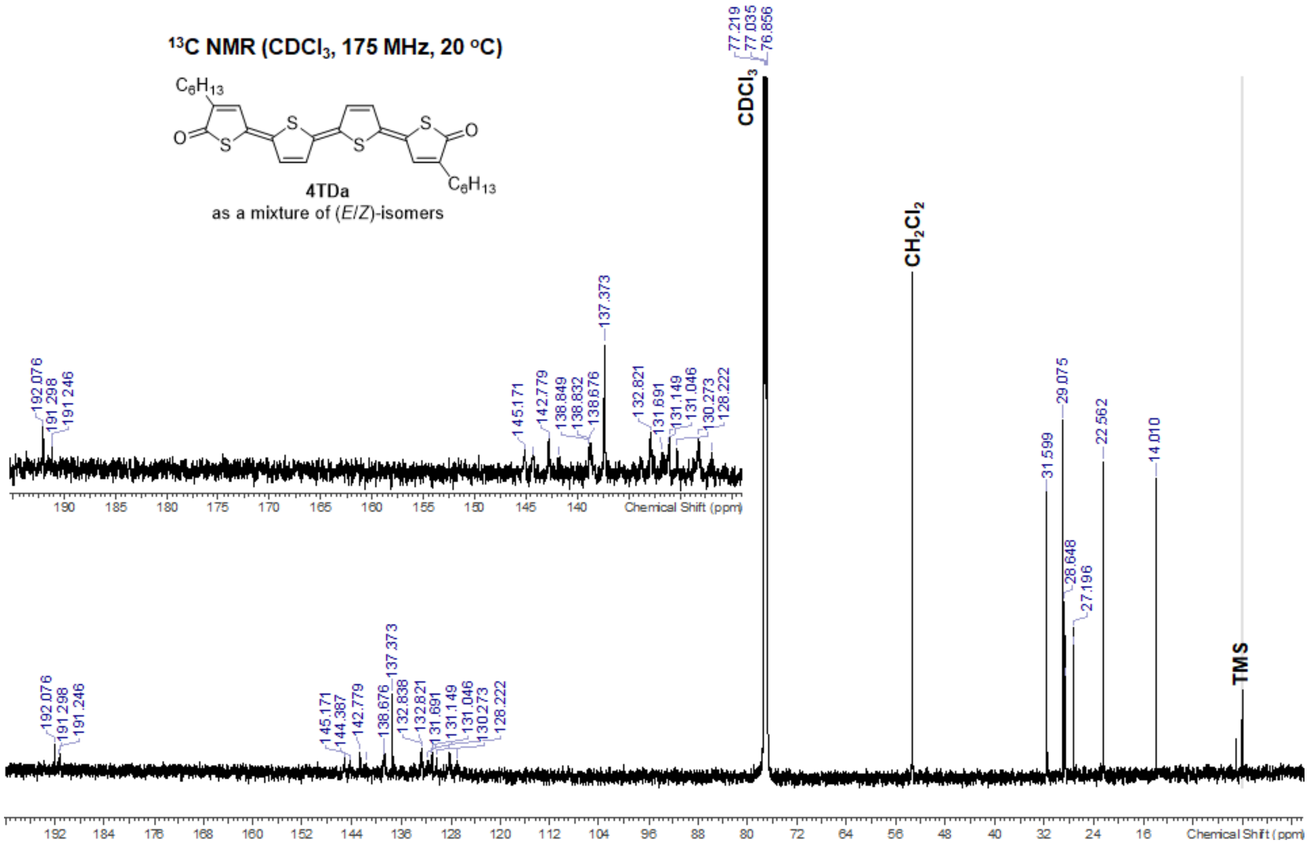

Figure S6. ^{13}C NMR spectra of 4TDa.

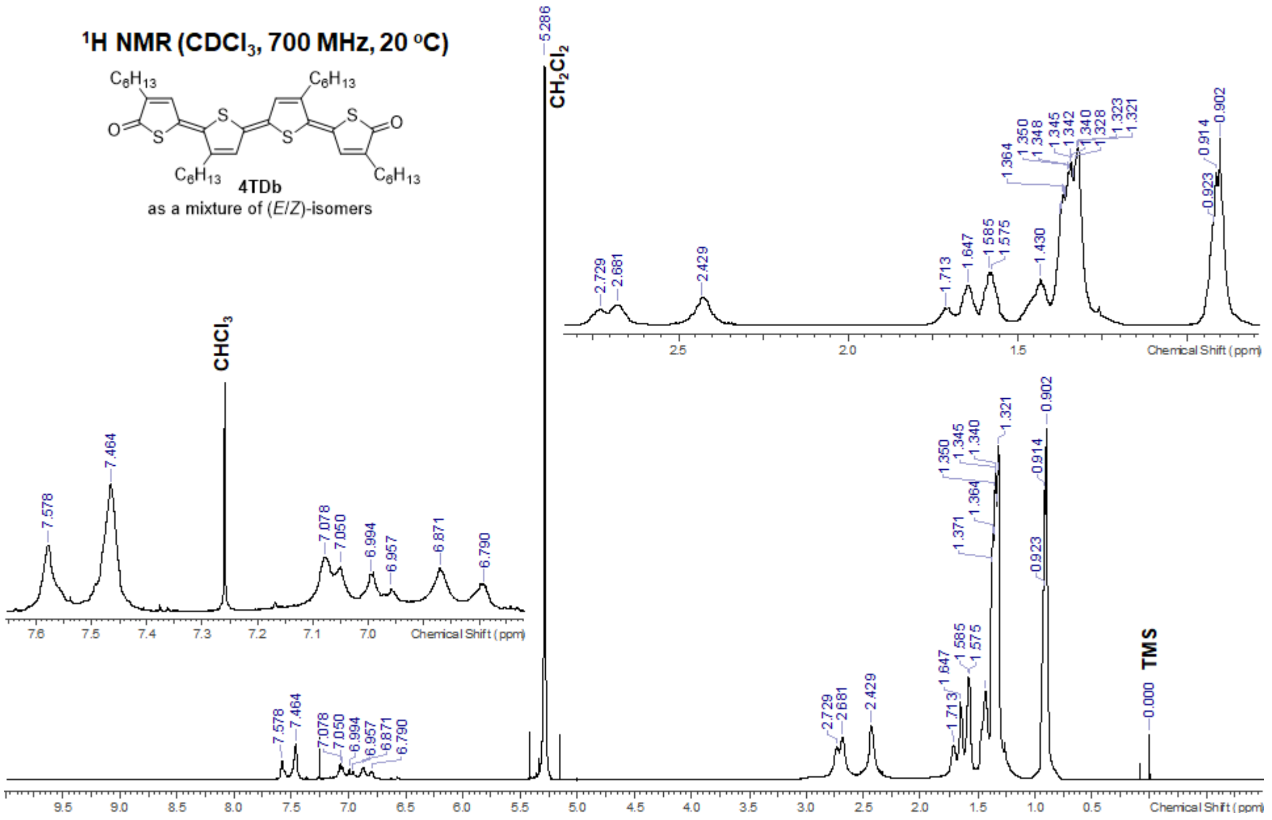

Figure S7. ^{1}H NMR spectra of 4TDb.

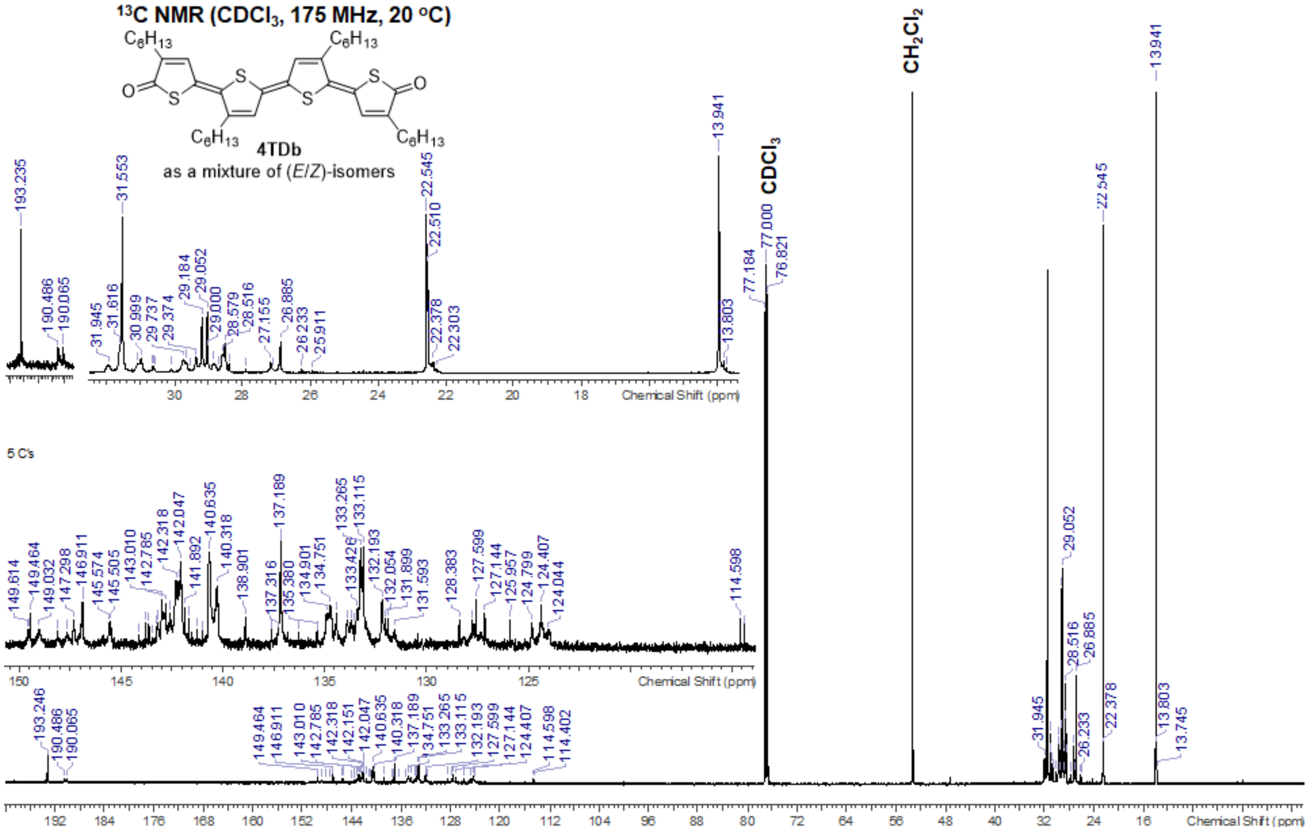

Figure S8. ^{13}C NMR spectra of 4TDb.

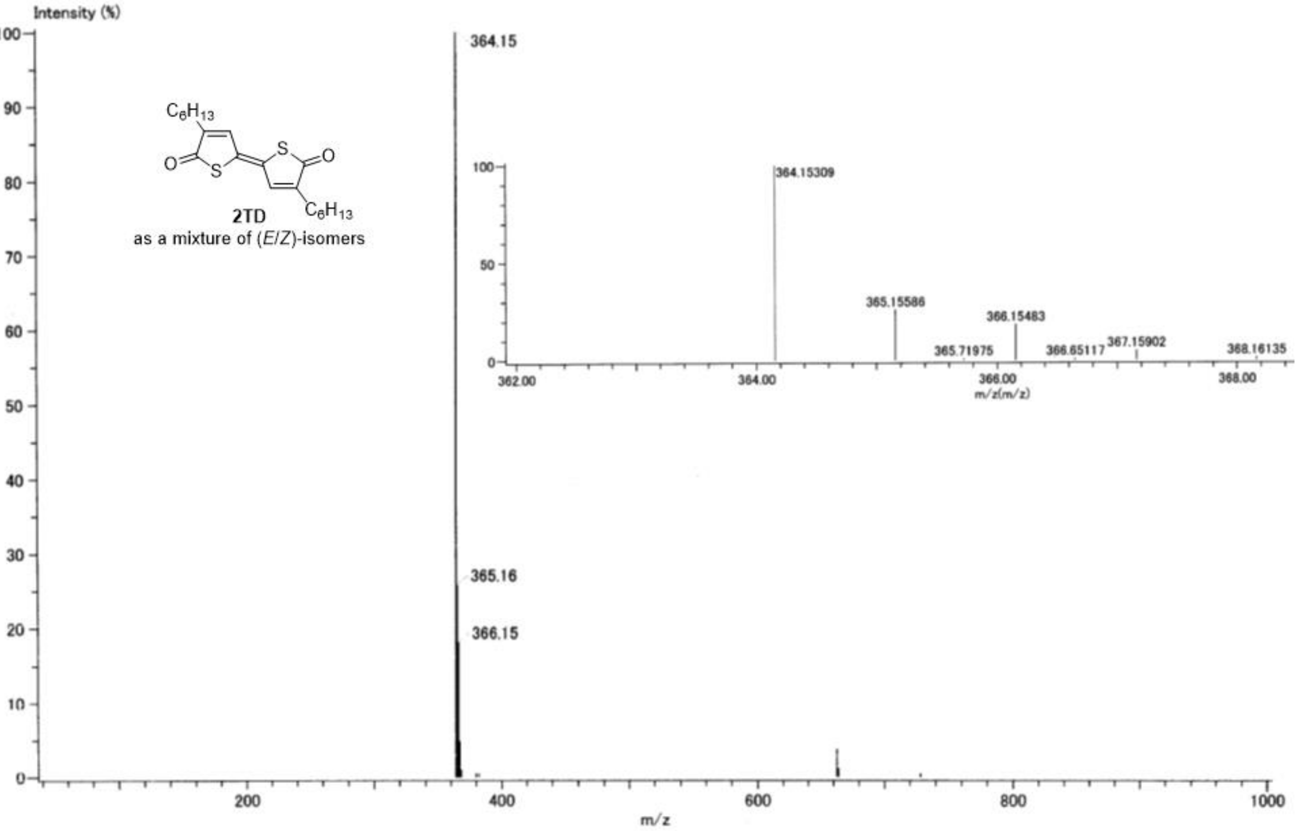

Figure S9. HRMS spectrum of 2TD.

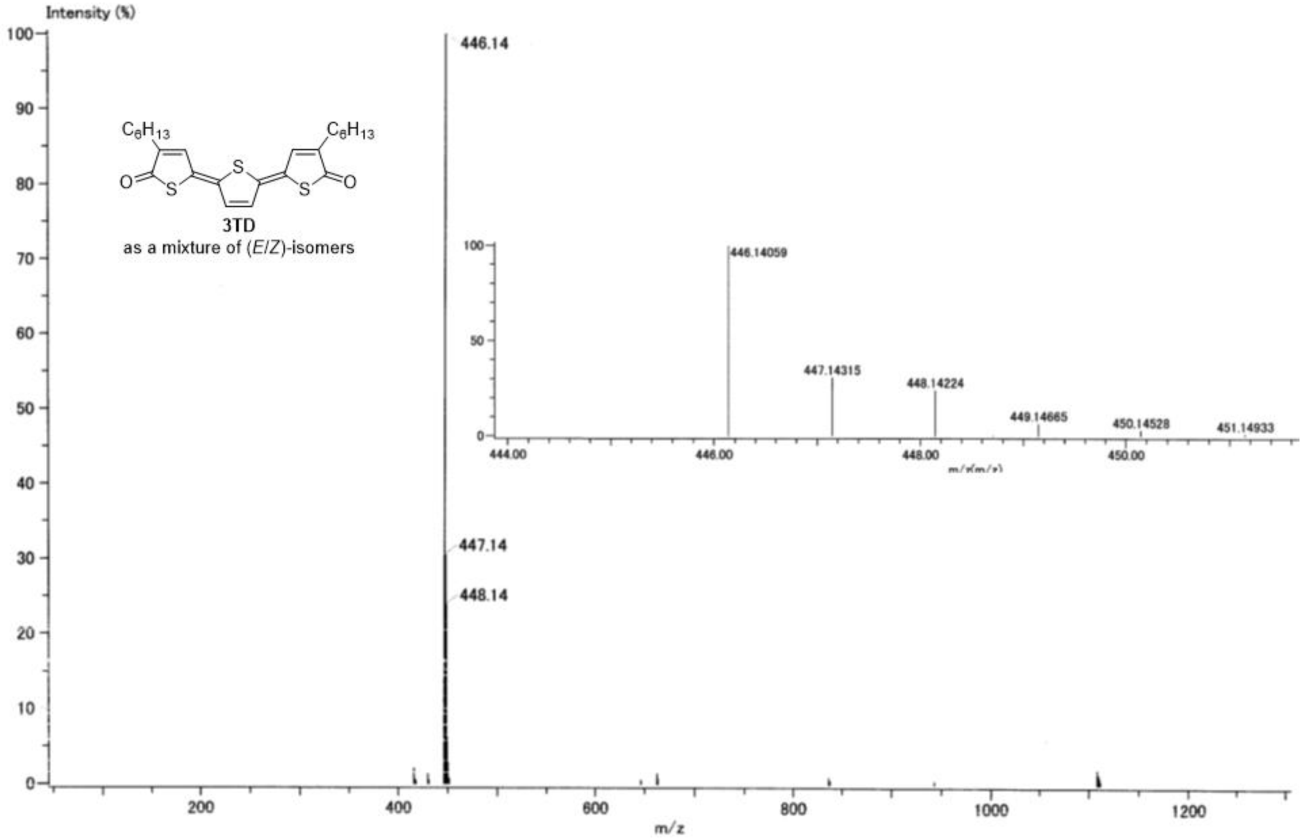

Figure S10. HRMS spectrum of 3TD.

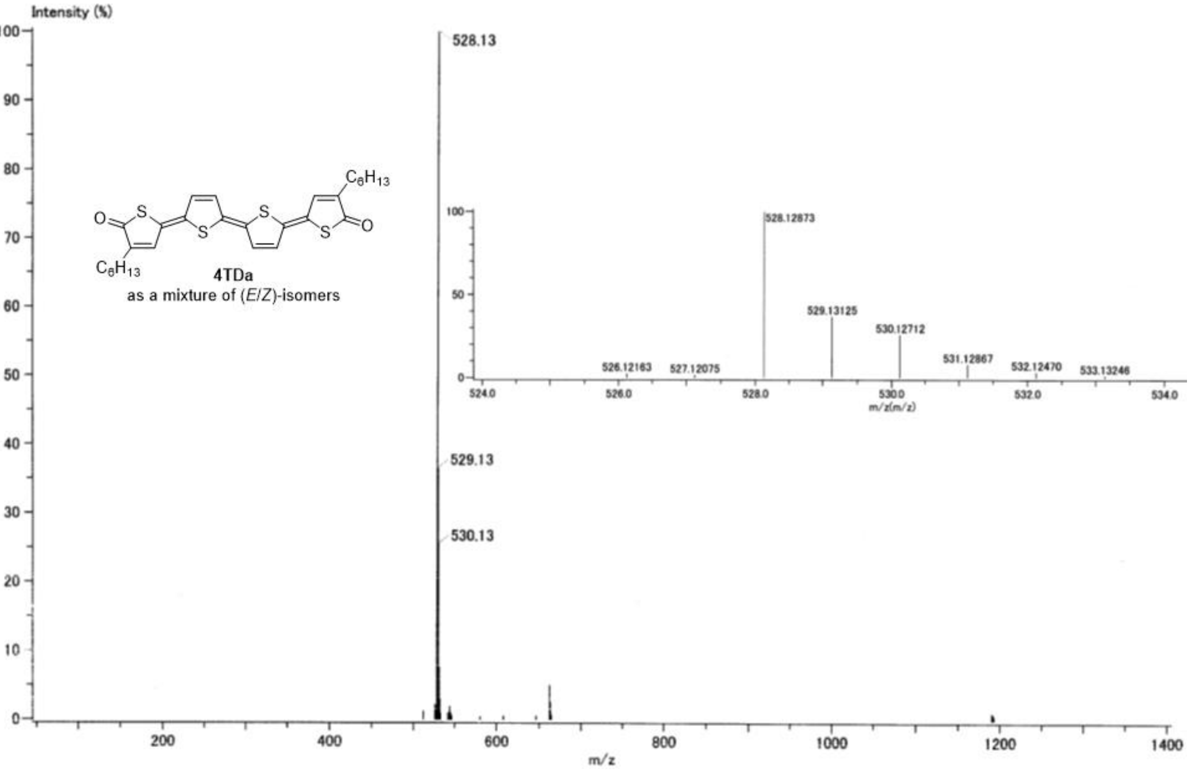

Figure S11. HRMS spectrum of 4TDa.

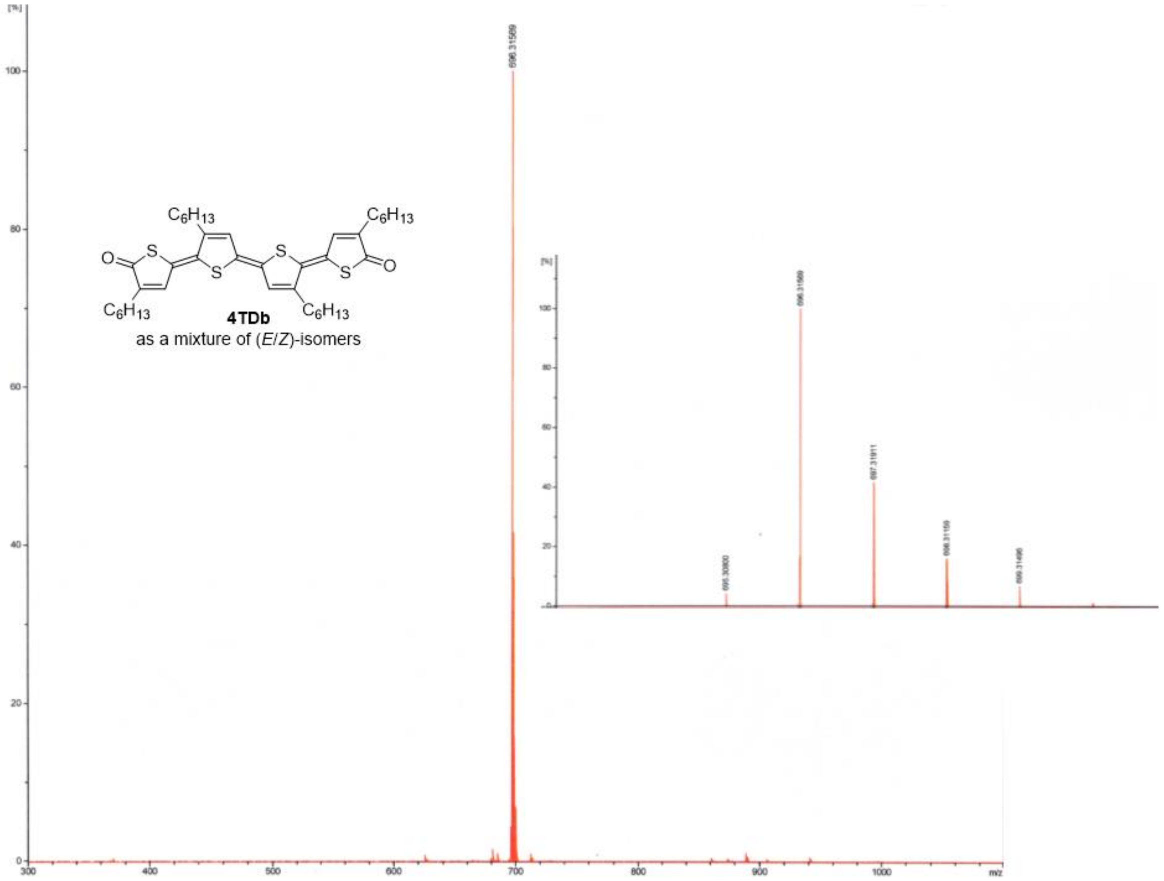

Figure S12. HRMS spectrum of 4TDb.

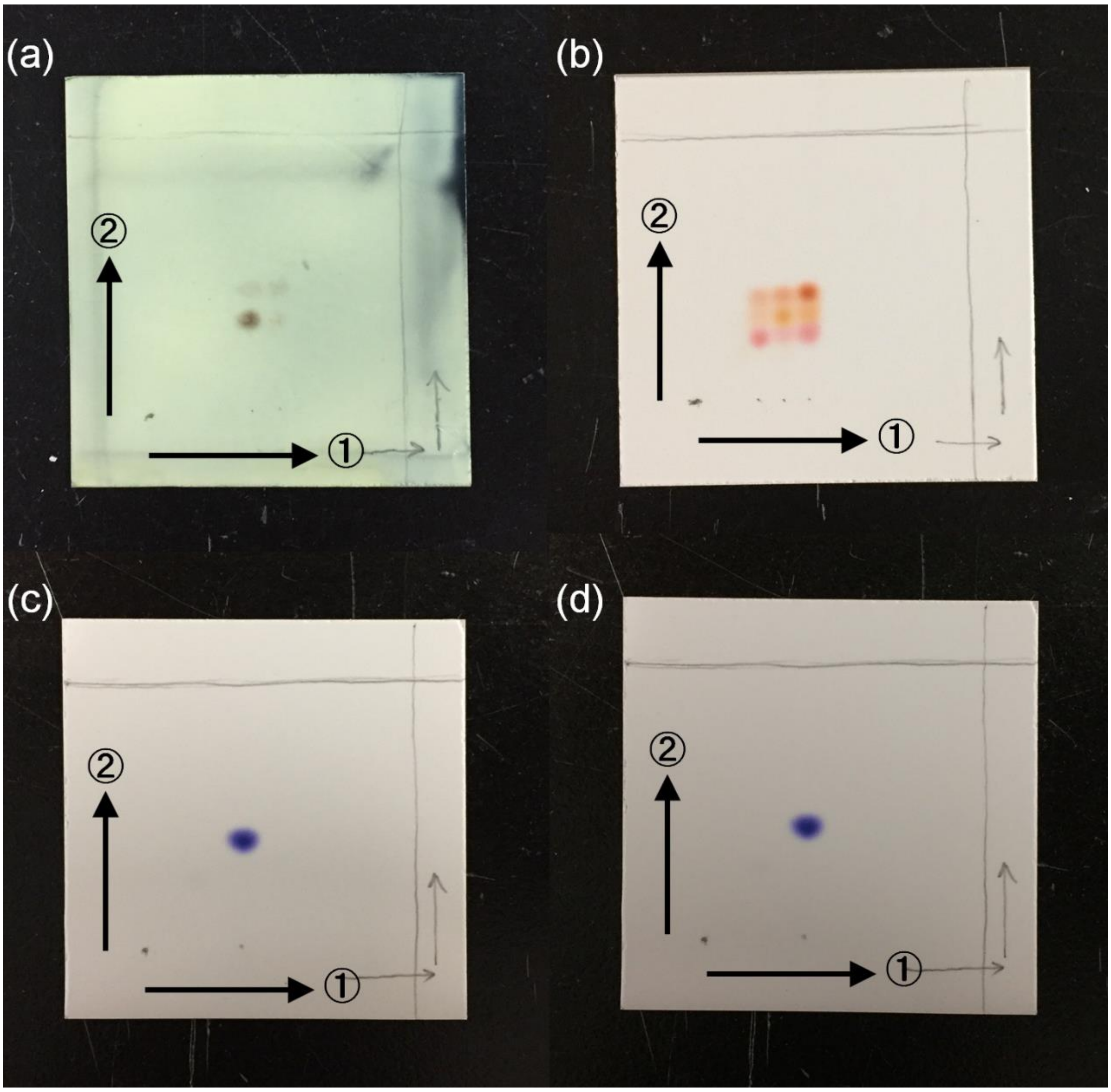

Figure S13. Photos of two-dimensional thin-layer chromatographic analyses for (**a**) 2TD with hexane: DCM = 5:5, which was stained with phosphomolybdic acid, (**b**) 3TD with hexane: DCM = 4:6, (**c**) 4TDa with hexane: DCM = 3:7, (**d**) 4TDb with hexane: DCM = 4:6.

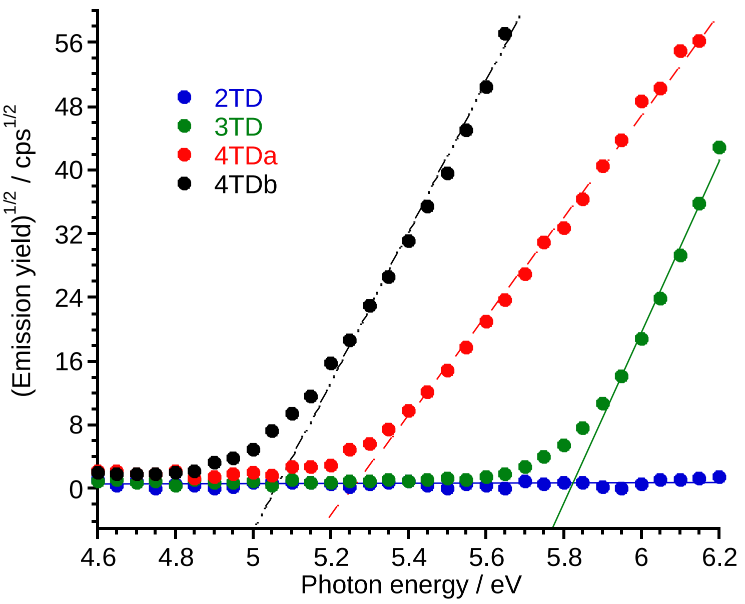

Figure S14. Photoemission yield spectra of thin films of 2TD, 3TD, 4TDa, and 4TDb.

Table S2. Crystallographic data for (*E*)-2TD (a) and (*ZEZ*)-4TDa.

**Compounds**	**(*E*)-2TD**	**(*ZEZ*)-4TDa**
Formula	$C_{20}H_{28}O_2S_2$	$C_{28}H_{32}O_2S_4$
Molecular weight	364.54	528.77
Crystal habit	platelet	block
Crystal system	monoclinic	triclinic
Space group	*C*2/*c*	*P*-1
a / Å	38.0998(9)	6.07816(16)
b / Å	4.08730(10)	6.27961(15)
c / Å	12.9588(3)	17.5127(4)
a / °	90	80.138(6)
b / °	108.0040(12)	85.649(6)
g / °	90	81.711(6)
V / $Å^3$	1919.2	650.78(3)
Temperature / K	100	100
Z	4	1
R, R_w	0.0662, 0.2379	0.0499, 0.1151
GOF	1.000	1.093

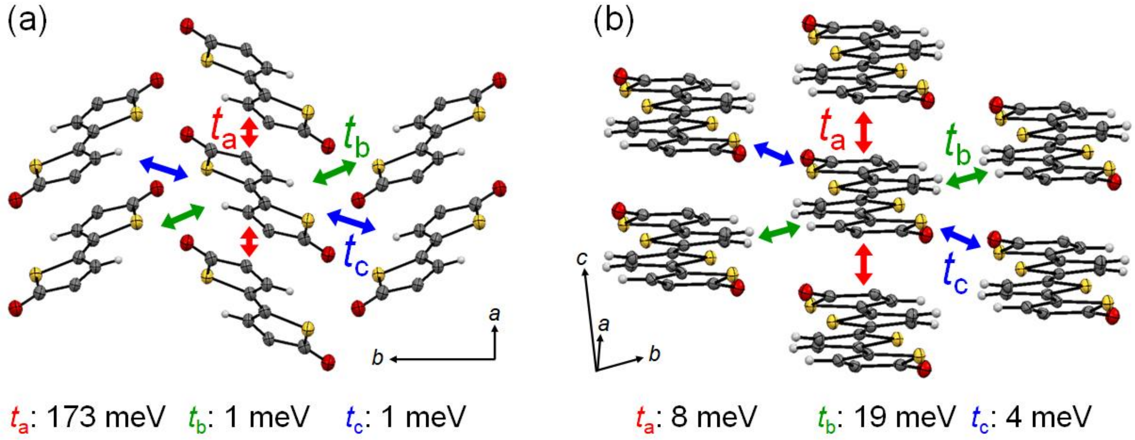

Figure S15. Packing structure and transfer integrals for electron transfer of (*E*)-2TD (a) and (*ZEZ*)-4TDa (b). Alkyl groups were omitted for clarity.

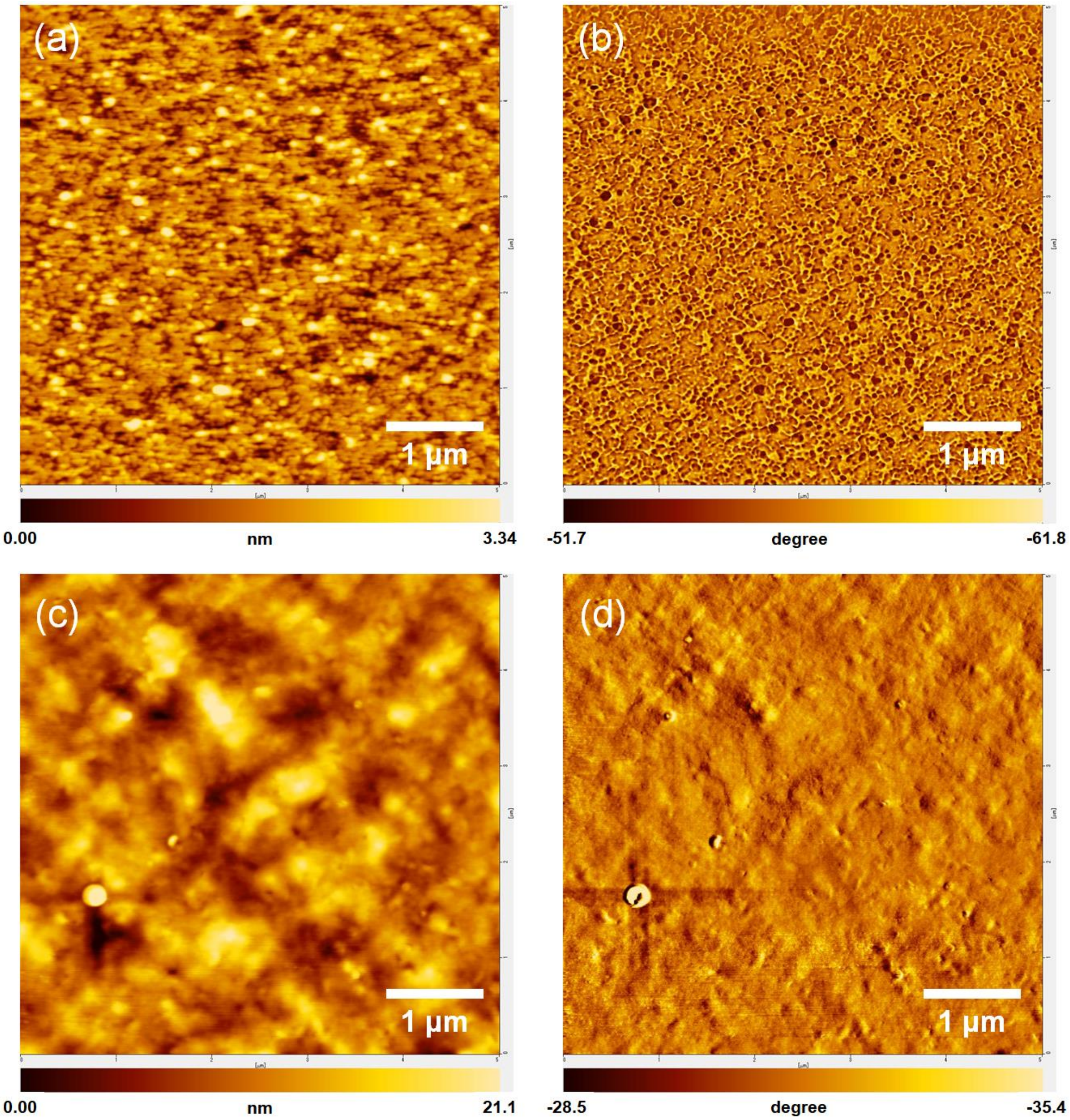

Figure S16. AFM height and phase images of the active layer in the OFET devices based on 4TDa (a and b) and 4TDb (c and d), respectively.

© 2020 by the authors. Licensee MDPI, Basel, Switzerland. This article is an open access article distributed under the terms and conditions of the Creative Commons Attribution (CC BY) license (http://creativecommons.org/licenses/by/4.0/).
